# Spontaneous episodic inflammation in the intestines of mice lacking HNF4A is driven by microbiota and associated with early life microbiota alterations

**DOI:** 10.1128/mbio.01504-23

**Published:** 2023-08-01

**Authors:** Cecelia Kelly, Jayanth Jawahar, Lauren Davey, Jeffrey I. Everitt, Joseph A. Galanko, Chelsea Anderson, Jonathan E. Avendano, Jessica R. McCann, R. Balfour Sartor, Raphael H. Valdivia, John F. Rawls

**Affiliations:** 1 Department of Molecular Genetics and Microbiology, Duke Microbiome Center, Duke University School of Medicine, Durham, North Carolina, USA; 2 Department of Immunology, Duke University School of Medicine, Durham, North Carolina, USA; 3 Department of Pathology, Research Animal Pathology Core, Duke University School of Medicine, Durham, North Carolina, USA; 4 Department of Medicine, Center for Gastrointestinal Biology and Disease, University of North Carolina at Chapel Hill, Chapel Hill, North Carolina, USA; Rutgers, The State University of New Jersey, New Brunswick, New Jersey, USA

**Keywords:** inflammatory bowel disease, gut microbiome, gut inflammation, HNF4A, NR2A1, longitudinal analysis, transcription factors

## Abstract

The inflammatory bowel diseases (IBD) occur in genetically susceptible individuals who mount inappropriate immune responses to their microbiota leading to chronic intestinal inflammation. Whereas IBD clinical presentation is well described, how interactions between microbiota and host genotype impact early subclinical stages of the disease remains unclear. The transcription factor hepatocyte nuclear factor 4 alpha (HNF4A) has been associated with human IBD, and deletion of *Hnf4a* in intestinal epithelial cells (IECs) in mice (*Hnf4a*^ΔIEC^) leads to spontaneous colonic inflammation by 6–12 mo of age. Here, we tested if pathology in *Hnf4a*^ΔIEC^ mice begins earlier in life and if microbiota contribute to that process. Longitudinal analysis revealed that *Hnf4a*^ΔIEC^ mice reared in specific pathogen-free (SPF) conditions develop episodic elevated fecal lipocalin 2 (Lcn2) and loose stools beginning by 4–5 wk of age. Lifetime cumulative Lcn2 levels correlated with histopathological features of colitis at 12 mo. Antibiotic and gnotobiotic tests showed that these phenotypes in *Hnf4a*^ΔIEC^ mice were dependent on microbiota. Fecal 16S rRNA gene sequencing in SPF *Hnf4a*^ΔIEC^ and control mice disclosed that genotype significantly contributed to differences in microbiota composition by 12 mo, and longitudinal analysis of the *Hnf4a*^ΔIEC^ mice with the highest lifetime cumulative Lcn2 revealed that microbial community differences emerged early in life when elevated fecal Lcn2 was first detected. These microbiota differences included enrichment of a novel phylogroup of *Akkermansia muciniphila* in *Hnf4a*^ΔIEC^ mice. We conclude that HNF4A functions in IEC to shape composition of the gut microbiota and protect against episodic inflammation induced by microbiota throughout the lifespan.

**IMPORTANCE:**

The inflammatory bowel diseases (IBD), characterized by chronic inflammation of the intestine, affect millions of people around the world. Although significant advances have been made in the clinical management of IBD, the early subclinical stages of IBD are not well defined and are difficult to study in humans. This work explores the subclinical stages of disease in mice lacking the IBD-associated transcription factor HNF4A in the intestinal epithelium. Whereas these mice do not develop overt disease until late in adulthood, we find that they display episodic intestinal inflammation, loose stools, and microbiota changes beginning in very early life stages. Using germ-free and antibiotic-treatment experiments, we reveal that intestinal inflammation in these mice was dependent on the presence of microbiota. These results suggest that interactions between host genotype and microbiota can drive early subclinical pathologies that precede the overt onset of IBD and describe a mouse model to explore those important processes.

## INTRODUCTION

The inflammatory bowel diseases (IBD), including Crohn’s disease (CD) and ulcerative colitis (UC), are spontaneously occurring chronic inflammatory diseases of the distal intestinal tract, which result from a combination of genetic susceptibility, immune system dysfunction, and environmental factors, including loss of homeostasis with the microbiota (1, 2). Clinical presentation of IBD can occur throughout the lifespan (3) and is associated with distinct pathophysiology and microbiota alterations (2, 4). Despite improvements in clinical approaches to IBD treatment in recent decades (5), the early subclinical or preclinical stages of IBD remain poorly understood and difficult to study (6, 7). An improved understanding of the subclinical stages of IBD could facilitate the development of preventative care screens and early intervention strategies for patients at risk of developing IBD.

Animal models provide opportunities to investigate subclinical IBD by permitting tissue-specific gene disruption and rigorous control over microbial colonization status. Mouse models that develop spontaneous colitis are particularly helpful, as they mimic the onset of human IBD. Causal contributions of microbiota to spontaneous intestinal inflammation have been demonstrated for many mouse IBD models (4, 8 - 12), while others still develop attenuated spontaneous intestinal inflammation under germ-free (GF) conditions (13 - 15). As IBD develops, the host’s homeostatic relationship with their microbiota may change. Some mouse models of IBD develop altered gut microbiota composition (16 - 20) while others may not (21). In some genetic models of IBD, their altered microbiotas are sufficient to confer disease phenotypes when transplanted into GF mouse recipients (22 - 24). Despite these utilities, one major limitation of many mouse models of IBD is that they develop disease relatively early in life and with high penetrance that does not reflect the typical IBD presentation observed in humans. Murine models of IBD that develop disease later in life with variable penetrance may present greater opportunities to discern early subclinical stages of disease before histological inflammation is apparent and symptoms such as weight loss, bloody stool, and rectal prolapse appear.

Intestinal epithelial cells (IECs) serve as a primary site of interaction between the host and microbiota. IECs form a barrier against the microbiota (25), sense their signals, and translate them into appropriate homeostatic responses (26). Genes involved in IEC biology, including transcriptional regulatory proteins, have been implicated in human IBD (27 - 29). HNF4A/NR2A1 is a nuclear receptor transcription factor expressed in the intestinal epithelium, with roles in IEC maturation and brush border formation (30), colonic ion transport (31), interactions with intra-epithelial lymphocytes (32), and prevention of spontaneous colitis (31) among other important functions (33 - 36). HNF4A has also been implicated in both major human IBD types, UC and CD. Non-coding single-nucleotide polymorphisms at *HNF4A* have been linked to UC (28, 29) and childhood onset CD (37). *HNF4A* mRNA expression is relatively reduced in intestinal tissues from CD and UC patients (38, 39), and HNF4A agonist treatment in mice upregulated many gene homologs downregulated in human IBD (40). HNF4A-binding sites in Caco2 cells are enriched for genetic variants associated with IBD risk (36), and HNF4A has been shown to regulate genes involved in immunity and inflammation in human cell lines and mice (27, 36). However, the role of *Hnf4a* in host-microbiota interactions had remained unknown. Combining genetic analysis and gnotobiotics in zebrafish, we discovered that most of the genes suppressed by microbiota colonization in the digestive tract are positively regulated by *hnf4a*. Furthermore, we found that microbial colonization in *hnf4a* mutant zebrafish yielded a transcriptional profile similar to human IBD (41). Additionally, we showed that microbiota colonization in mice reduced HNF4A chromatin occupancy in small intestinal IECs (41). Together these findings suggested that microbiota may promote intestinal inflammation in HNF4A-deficient hosts and that interactions between microbiota and HNF4A activity in the epithelium are required to maintain gut homeostasis. Mice with IEC-specific knockout of HNF4A (*Hnf4a*^ΔIEC^ mice) display increased susceptibility to intestinal injury with dextran sodium sulfate (32, 36, 38). However, spontaneous colitis in *Hnf4a*^ΔIEC^ mice has been described in only one study, which reported severe and fully penetrant colonic inflammation by 6–12 mo of age (31). This is a significantly longer disease development timeframe than many other well-studied genetic (4, 16, 24, 42 - 46) and chemically induced mouse models of IBD (47 - 49), presenting an interesting opportunity to study how inflammation and host-microbiota relationships evolve over time in the subclinical stages leading up to spontaneous colitis. Here we characterized the dynamic progression of disease onset and gut microbial ecology in *Hnf4a*^ΔIEC^ mice. We also address the question of whether microbiota are required for the development of spontaneous colitis in *Hnf4a*^ΔIEC^ mice. Our findings indicate that *Hnf4a*^ΔIEC^ mice can serve as a useful model to study subclinical stages of IBD development.

## RESULTS

### *Hnf4a*^ΔIEC^ mice exhibit episodically elevated fecal Lcn2 and episodic loose stools starting early in life, which worsen with age in a subset of animals

**Fig 1:**
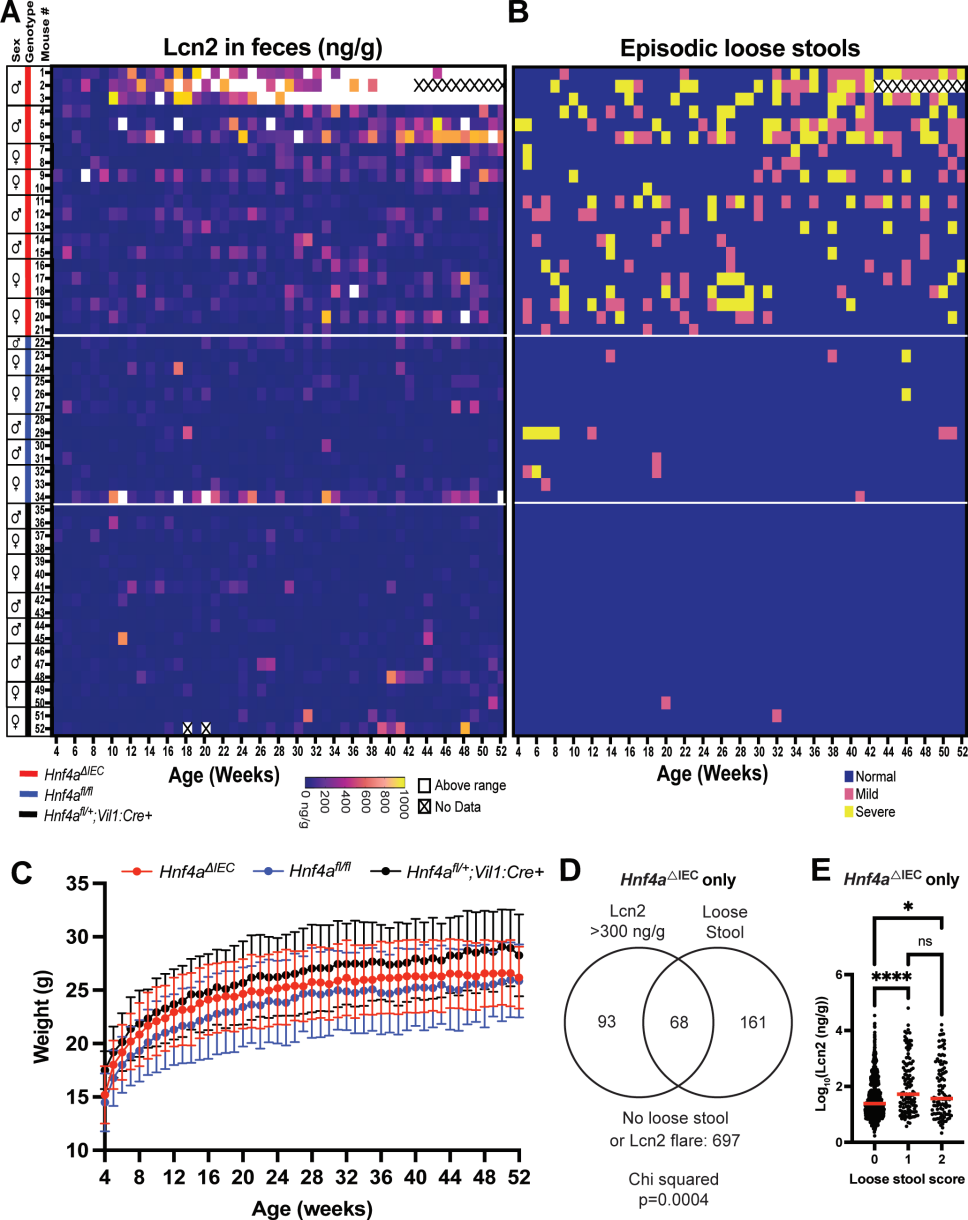
Longitudinal phenotypic assessment of SPF *Hnf4a*^ΔIEC^ mice. (**A**) Heatmap of combined data from two independent experiments showing fecal Lcn2 at weekly timepoints in individual *Hnf4a*^ΔIEC^ and control mice. Cage is denoted by black box on left enclosing sex, genotype, and mouse # information. Note that mouse 2 was euthanized early at 42 wk due to an irreducible rectal prolapse. (**B**) Heatmap showing episodic loose stool incidence. (**C**) Average weight of all mice in each group. (**D**) Distribution of Lcn2 >300 ng/g, loose stools, and their overlap in *Hnf4a*^ΔIEC^ mice. (**E**) Comparison of Log_10_(Lcn2) by loose stool score. *P*-values for (**E**) calculated using Kruskal-Wallis test followed by Dunn’s multiple comparisons test. ****P < 0.0001, *P < 0.05, ns = not significant.

We sought to define the timeframe in which initial inflammatory events occur in the gut of *Hnf4a*^ΔIEC^ mice. To evaluate gut inflammation longitudinally in a non-invasive manner, we measured fecal lipocalin 2 (Lcn2), a sensitive biomarker of gut inflammation in mice (50) and humans (51). We tested whether Lcn2 was associated with genotype, sex, age, and genotype by age interaction using a repeated measures regression model. We found that genotype was a predictor of Lcn2 levels (*P* = 0.0002), with *Hnf4a*^ΔIEC^ showing higher Lcn2 levels compared to *Hnf4a*^fl/fl^ (*P* = 0.018) and *Hnf4a*^fl/+^;*Vil1:Cre+* mice (*P* = 0.0063) (Fig. 1A). Age (*P* < 0.0001) and genotype by age interaction (*P* = 0.0034) were both predictors of Lcn2 levels, but sex was not (*P* = 0.3590). *Hnf4a*^ΔIEC^ mice had consistently significantly elevated Lcn2 levels compared to *Hnf4a*^fl/fl^ mice starting at 21 wk of age, while *Hnf4a*^fl/+^;*Vil1:Cre+* mice did not (Fig. S1A). Together this suggests that *Hnf4a*^ΔIEC^ mice had more age-dependent intestinal inflammation than sibling controls (*Hnf4a*^fl/fl^ and *Hnf4a*^fl/+^;*Vil1:Cre+* mice). Intriguingly, inflammation in *Hnf4a*^ΔIEC^ mice appeared to be episodic, exhibiting a flaring pattern months before becoming chronically elevated in a subset of mice (Fig. 1A). Based on previous studies in other mouse IBD models that related fecal Lcn2 to histopathology (50, 52), we operationally defined Lcn2 levels above 300 ng/g of feces as indicative of inflammation. We speculate that early episodically elevated Lcn2 represents a transient inflammatory response in the gut that is self-resolving, perhaps caused by microinjury of the intestine or a transient microbial infection.

*Hnf4a*^ΔIEC^ mice also displayed episodic loose stools between 4 and 20 wk of age (Fig. 1B; Fig. S1B). Using a repeated measures regression analysis, we found that episodic loose stool incidence was higher in *Hnf4a*^ΔIEC^ compared to control mice (*Hnf4a*^fl/fl^ and *Hnf4a*^fl/+^;*Vil1:Cre+* mice) (*P* < 0.0001) (Fig. 1B). Episodic loose stool incidence was biased toward males in *Hnf4a*^ΔIEC^ mice (*P* = 0.0406), but this sex bias was not observed in controls (*P* = 0.749). Age was not a predictor of episodic loose stool incidence (*P* = 0.3028). Together these results suggest that *Hnf4a*^ΔIEC^ mice develop episodic loose stools that are age independent and more likely to occur in males.

Next, we tested whether body weight was associated with genotype, sex, and age with a repeated measures regression model. We found that *Hnf4a*^ΔIEC^ mouse body weight was not different from *Hnf4a*^fl/fl^ controls (*P* = 0.2896) (Fig. 1C). Age in weeks (*P* < 0.0001) and sex (*P* < 0.0001) were predictors of weight. Unexpectedly, *Hnf4a*^fl/+^;*Vil1:Cre+* mice weighed more than both *Hnf4a*^fl/fl^ (*P* < 0.0001) and *Hnf4a*^ΔIEC^ mice (*P* = 0.0013) (Fig. 1C), but those differences were not further explored here.

Lastly, we tested whether incidences where Lcn2 >300 ng/g and episodes of loose stools were temporally coupled. A chi-squared test revealed that the number of timepoints at which *Hnf4a*^ΔIEC^ mice displayed fecal Lcn2 >300 ng/g and loose stools was more frequent than expected by chance (*P* = 0.0004), assuming the incidences of each phenomenon were equally likely at any given timepoint within the *Hnf4a*^ΔIEC^ group (Fig. 1D). Additionally, when *Hnf4a*^ΔIEC^ mice had loose stools, they tended to have significantly higher Lcn2 values (Fig. 1E). Overall, this suggests that episodic loose stools are associated with episodically elevated fecal Lcn2, but the two phenotypes can also occur independently of each other.

### *Hnf4a*^ΔIEC^ mice develop mild histopathological features of colitis by 12 mo of age

**Fig 2:**
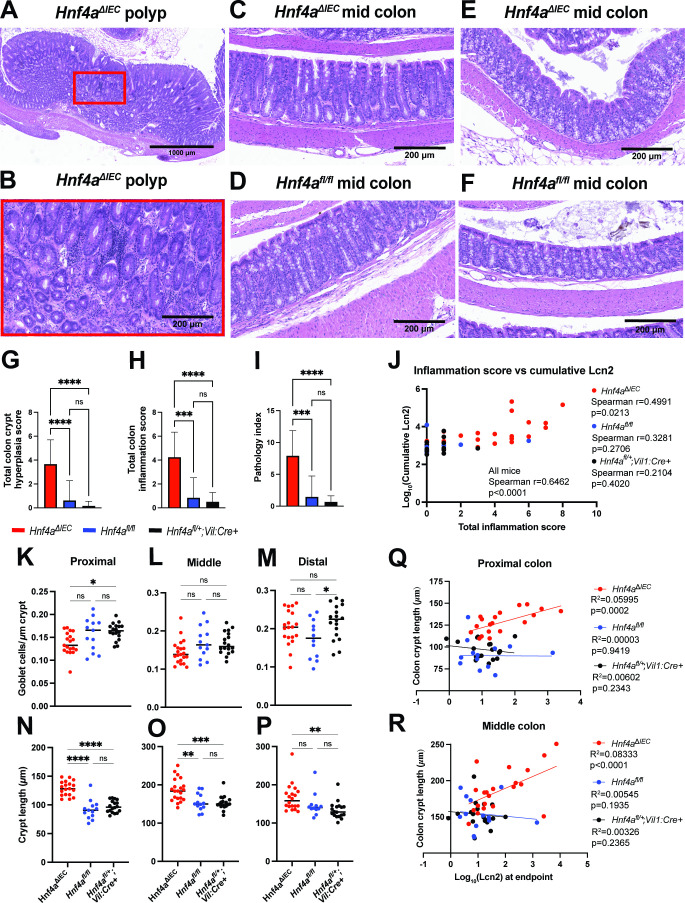
Assessment of colon histopathology in 52-wk-old SPF *Hnf4a*^ΔIEC^ mice. (**A**) Representative image of colon polyp. (**B**) Magnification showing inflammatory infiltrates inside the polyp. (**C**) Representative image of crypt elongation in *Hnf4a*^ΔIEC^ mice. (**D**) Representative image of crypt elongation in control mice. (**E**) Representative image of less elongated crypts in *Hnf4a*^ΔIEC^ mice. (**F**) Representative image of normal colon epithelium in control mice. (**G–I**) Average histopathological scores. (**J**) Spearman correlation between log_10_(cumulative Lcn2) and total inflammation score. Spearman *r* and *P*-values for all mice as well as individual genotypes are indicated. (**K–P**) Average measurements of goblet cell/μm crypt and crypt length in different colon segments. (**Q–R**) Simple linear regression showing a positive relationship between colon crypt length and log_10_(Lcn2) at endpoint in *Hnf4a*^ΔIEC^ mice but not controls. *P*-values for (**G–I, L–P**) were calculated using Kruskal-Wallis test followed by Dunn’s multiple comparisons test, and (**K**) using one-way ANOVA followed by Holm-Sidak’s multiple comparisons testing. ****P < 0.0001, ***P < 0.001, **P < 0.01, *P < 0.05, ns = not significant.

A previous study reported severe, fully penetrant colonic inflammation in SPF *Hnf4a*^ΔIEC^ mice by 12 mo of age (31). We observed a much milder histopathological phenotype in our SPF *Hnf4a*^ΔIEC^ mice at 12 mo of age, characterized by mild-to-moderate crypt hyperplasia, mild goblet cell loss, and inflammatory infiltrate composed primarily of lymphocytes with a small number of plasma cells admixed (Fig. 2A through C and E). Occasionally, scattered neutrophils were also present but never in clusters. These infiltrates were limited to the lamina propria of the mucosa in almost all cases. We did not perform histopathological evaluation of the cecum in this study. Together, we interpret these features as mild histopathological features of colitis (Fig. 2A through F). These features were discontinuous across the colon and varied in severity between mice. In 2/21 SPF *Hnf4a*^ΔIEC^ mice examined, we observed colonic polyps (Fig. 2A and B), in agreement with the previous report (31). We observed significantly elevated goblet cell numbers in the ileum of *Hnf4a*^ΔIEC^ mice (Fig. S1C) but found no evidence of inflammation in the small intestine.

It was difficult to distinguish *Hnf4a*^ΔIEC^ mice from controls with high accuracy due to the subtle phenotype (Fig. 2C through F), so we paired blind histopathological scoring with quantitative assessments of goblet cell number and crypt length to further assess the phenotype. We found that total colon crypt hyperplasia and inflammation scores, as well as pathology index (which we operationally define as the sum of crypt hyperplasia and inflammation scores), were all significantly higher in *Hnf4a*^ΔIEC^ mice compared to controls (Fig. 2G through I). We also found that these scores had a significant, positive correlation with cumulative lifetime fecal Lcn2 for all mice and for the *Hnf4a*^ΔIEC^ group alone but not for individual control groups (Fig. 2J). Quantitation of the number of goblet cells/crypt showed a significant decrease in the proximal colon in *Hnf4a*^ΔIEC^ mice compared to *Hnf4a*^fl/+^;*Vil1:Cre+* mice and a similar trend compared to *Hnf4a*^fl/fl^ mice (*P* = 0.085) (Fig. 2K through M). Quantitative measurements of colon crypt length show an increase in *Hnf4a*^ΔIEC^ mice compared to controls, suggesting increased colon epithelial proliferation (Fig. 2N through P). Simple linear regression analysis showed a modest but significant positive relationship between endpoint log_10_(Lcn2) values and colon crypt length in *Hnf4a*^ΔIEC^ mice but not control groups (Fig. 2Q and R). These results establish that *Hnf4a*^ΔIEC^ mice develop mild, variable histopathological features of colitis by 12 mo of age.

### Antibiotic depletion of microbiota reduced Lcn2 levels and episodic loose stool incidence in *Hnf4a*^ΔIEC^ mice

**Fig 3:**
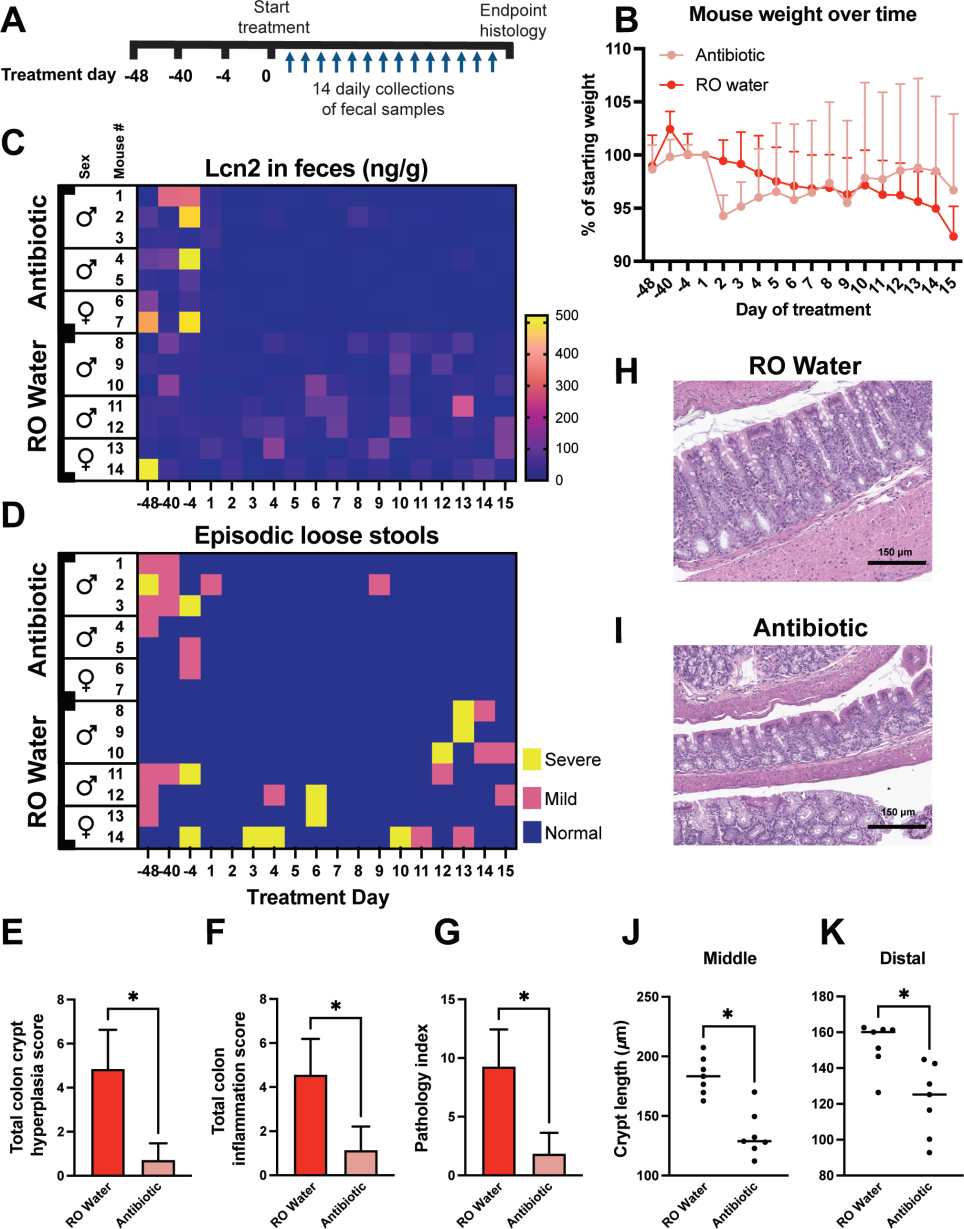
Treatment of *Hnf4a*^ΔIEC^ mice with antibiotics. (**A**) Treatment and sampling timeline. (**B**) Average % starting weight of mice in each group. (**C**) Heatmap showing fecal Lcn2 at three pretreatment timepoints and daily timepoints after treatment started in antibiotic and mock-treated *Hnf4a*^ΔIEC^ mice. (**D**) Heatmap showing episodic loose stool incidence at same timepoints as (**C**). (**E–F**) Average colon histopathological scores. (**H–I**) Representative images of colon crypt length in the two groups. (**J–K**) Average crypt length in middle and distal colon. *P*-values for (**G–J**) were calculated using a two-tailed Wilcoxon matched-pairs signed rank test and (**K**) using a two-tailed paired *t* test. *P < 0.05.

To test the role of microbiota on gut inflammation and loose stools in SPF *Hnf4a*^ΔIEC^ mice, we depleted the microbiota by administering broad-spectrum antibiotics (ampicillin, vancomycin, neomycin, metronidazole, and fluconazole; see Supplemental methods). Increased fecal Lcn2 and episodic loose stool phenotypes in SPF *Hnf4a*^ΔIEC^ mice were confirmed at three timepoints prior to the beginning of antibiotic treatment (Fig. 3A). Antibiotic treatment resulted in the depletion of bacteria by 3 d as measured by quantitative PCR for the bacterial 16S rRNA gene in fecal DNA preparations (Fig. S1D). Phenotypic assessments over 14 d of antibiotic or reverse osmosis (RO) purified water control treatment (Fig. 3A) showed the body weight of mice treated with antibiotics was not significantly different from controls (*P* = 0.2601) (Fig. 3B), but treated mice had significantly lower Lcn2 levels (*P* < 0.0001) (Fig. 3C). Sex did not have a significant effect on Lcn2 levels (*P* = 0.2975). Antibiotic treated mice also had reduced episodic loose stool frequency (Fig. 3D), total colon crypt hyperplasia and inflammation scores, and combined pathology index (Fig. 3E through G). Middle and distal colon crypt length were also reduced (Fig. 3H through K), while proximal colon crypt length and goblet cell numbers were not impacted (data not shown). Together these results suggest that the elevated fecal Lcn2, episodic loose stools, and crypt elongation phenotypes in SPF *Hnf4a*^ΔIEC^ mice can be rescued by depleting microbiota.

### *Hnf4a*^ΔIEC^ mice have significantly reduced Lcn2 levels when raised germ free

To more rigorously define the impact of microbiota on the phenotypes of *Hnf4a*^ΔIEC^ mice, we derived the *Hnf4a*^fl/fl^ and *Hnf4a*^fl/+^;*Vil1:Cre+* mouse lines into GF conditions. HNF4A is generally thought to be a transcriptional activator (36, 53, 54), and our prior results suggested that HNF4A promotes expression of genes that are downregulated by microbiota, many of which are also downregulated in human IBD (41). We hypothesized that *Hnf4a*^ΔIEC^ mice reared in the absence of microbiota would not develop colitis, episodically elevated fecal Lcn2, or episodic loose stools.

**Fig 4:**
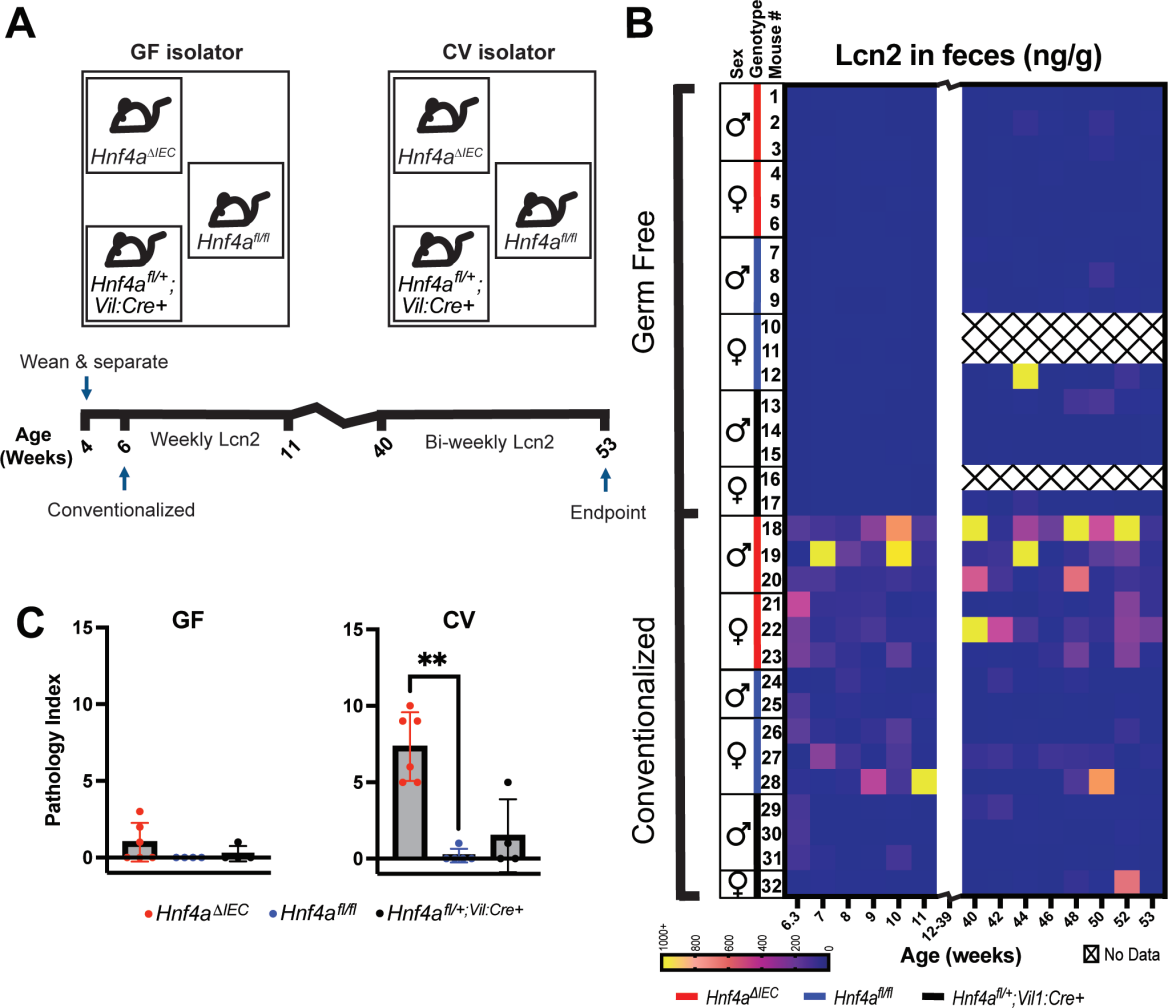
Comparison of GF and CV *Hnf4a*^ΔIEC^ and control mice. (**A**) Schematic of gnotobiotic isolators and experimental timeline. (**B**) Heatmap showing Lcn2 at weekly and biweekly timepoints in early and late life stages of experimental mouse groups. Cage is denoted by black boxes on left enclosing sex, genotype, and mouse # information. Note that mice 10, 11, and 16 were found dead prior to endpoint (cause undetermined). (**C**) Average histopathological scores of mice at experimental endpoint. *P*-values for (**C**) were calculated using two Kruskal-Wallis tests with Dunn’s multiple comparisons testing. One test was performed per microbial condition to compare the three genotypes within that microbial condition. **P < 0.01.

GF *Hnf4a*^ΔIEC^ mice were born at expected Mendelian ratios (Fig. S1E) and did not exhibit excess morbidity or mortality compared to GF and CV controls or CV *Hnf4a*^ΔIEC^ mice. We generated a cohort of 12 *Hnf4a*^ΔIEC^, 11 *Hnf4a*^fl/fl^, and 9 *Hnf4a*^fl/+^;*Vil1:Cre+* mice and maintained half of them in GF conditions, while the other half were conventionalized at 6 wk of age with microbiota collected from SPF control mice. Fecal Lcn2 and episodic loose stools were tracked at early life (week 6–11) and later life (week 40–53) timepoints, and colon histopathology was assessed at 53 wk (Fig. 4A and B). We ran a repeated measures regression model to test whether Lcn2 was associated with genotype, colonization status, and life stage (Table S1). Consistent with our SPF experiments, Lcn2 levels were significantly higher in CV *Hnf4a*^ΔIEC^ compared to CV controls (Fig. 4B; Table S1). In contrast, GF *Hnf4a*^ΔIEC^ mice did not have significantly higher Lcn2 compared to GF controls. Lcn2 levels were also elevated in CV compared to GF mice in both control genotypes, confirming the proinflammatory impact of microbiota even in wild-type animals. Finally, CV *Hnf4a*^ΔIEC^ mice showed significantly higher Lcn2 compared to GF *Hnf4a*^ΔIEC^ mice. Colon pathology index in CV *Hnf4a*^ΔIEC^ mice was significantly higher than CV controls but was similar between GF *Hnf4a*^ΔIEC^ and GF controls (Fig. 4C; see representative images in Fig. S2). Together these results establish that microbiota are required for the episodically elevated fecal Lcn2 and colon pathology index increases we observed in *Hnf4a*^ΔIEC^ mice.

Some phenotypic aspects observed in our SPF experiments described earlier (Fig. 1 and 2) were not observed under gnotobiotic conditions. Quantification of crypt length in all three genotypes revealed that microbiota colonization increased crypt length in the middle colon with a similar pattern in the distal colon (Fig. S3A through C). Quantification of goblet cell number in the colon revealed no consistent effect of microbial colonization status (Fig. S3D through F), and neither colon crypt length nor goblet cell number revealed any significant interaction between colonization status and *Hnf4a* genotype (Fig. S3A through F). Unlike our SPF experiments earlier (Fig. 1 and 3), gnotobiotic GF and CV mice of all genotypes displayed very few incidences of loose stools, preventing conclusions about the role of microbiota in episodic loose stools. The causes for these phenotypic differences under SPF and gnotobiotic conditions here are unknown but could include differences in facility environment, mouse and sample handling procedures, host age and timing/duration of colonization, and diet or inoculating microbiota composition.

### Intestinal epithelial *Hnf4a* regulates gut microbiota composition at 52 wk

**Fig 5:**
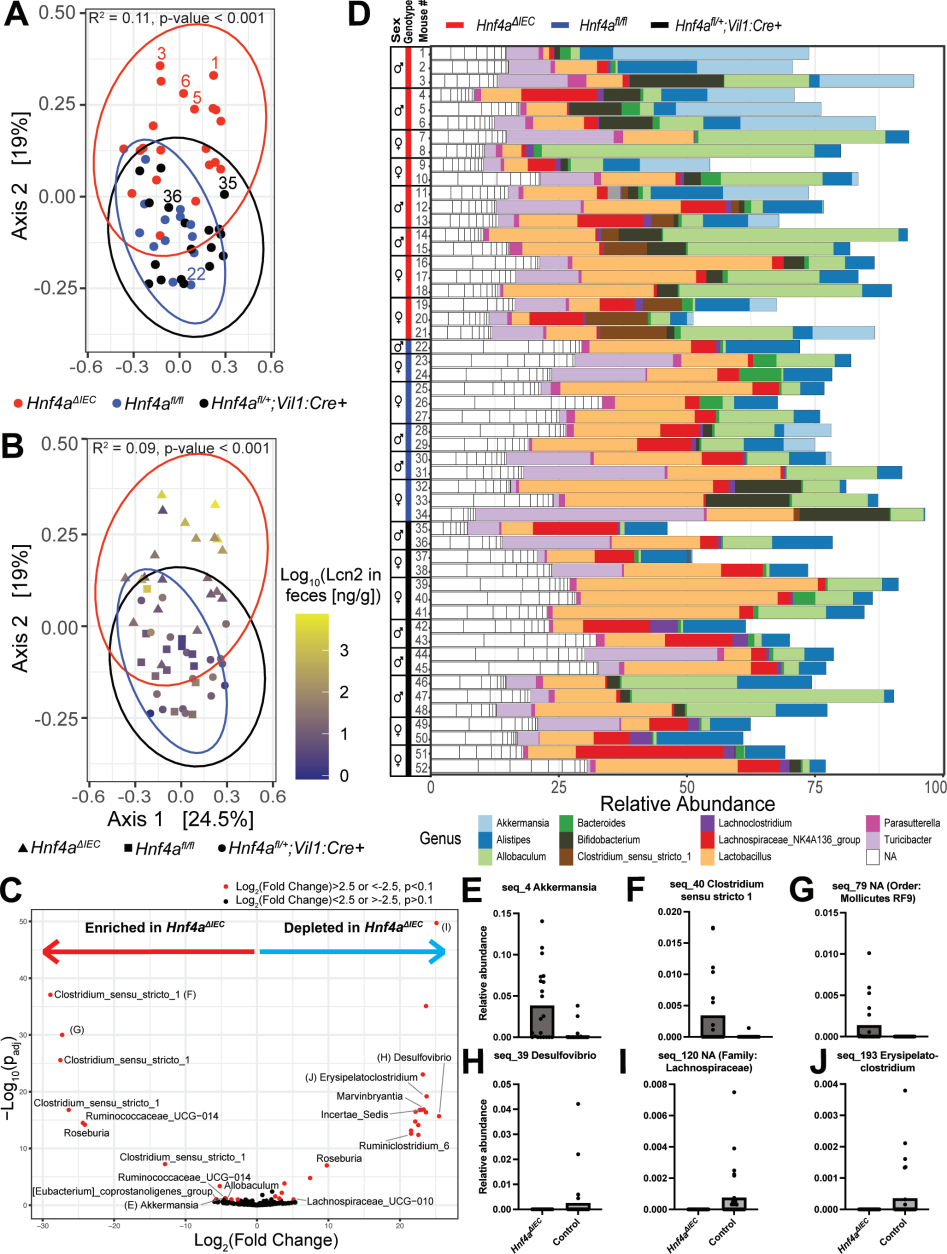
Fecal microbiome composition in SPF *Hnf4a*^ΔIEC^ mice and controls at 52 wk of age. (**A–B**) PCoA of Bray-Curtis dissimilarity colored by (**A**) genotype and (**B**) log_10_(Lcn2) levels. 95% Confidence ellipses are color coded to genotype. Mice selected for further longitudinal analysis in Fig. 6 are labeled with numbers in (**A**). PERMANOVA *R*^2^ and *P-*values are indicated at the top of panels A and B, showing the percentage of variance explained by genotype and log_10_(Lcn2), respectively. (**C**) Volcano plot showing differentially enriched ASVs between *Hnf4a*^ΔIEC^ mice and controls. Assigned genus is labeled on points with log_2_(fold change) >4 or <−4 and *p*_adj_ <0.1. Points selected for representative graphing in panels (**E–J**) are indicated with corresponding panel letter on volcano plot. (**D**) Relative abundance in each sample of the top 25 most abundant genera in the data set. Genera labeled “NA” were not assigned taxonomic information at the genus level. (**E–J**) Representative graphs showing relative abundance of differentially enriched ASVs (see also Fig. S4E and F and Table S2). Note that “seq_#” indicates the most abundant ASVs in the data set in descending order (e.g., “seq_4 Akkermansia” is the fourth most abundant ASV in the data set). All fecal samples here were from 52 wk of age except mouse 2, which was collected at 42 wk when it was euthanized.

To determine if intestinal microbiota composition was altered in *Hnf4a*^ΔIEC^ mice, we profiled 16S rRNA gene sequences amplified from fecal samples collected during our SPF mouse experiments (Fig. 1) at 52 wk. Multiple indices of alpha diversity (Shannon, Chao1, and Simpson) were not significantly different between the *Hnf4a*^ΔIEC^ mice and controls (data not shown). However, permutational analysis of variance (PERMANOVA) of Bray-Curtis and weighted UniFrac distances between samples revealed that genotype had a significant effect on microbiota composition, accounting for 11.2% (*P* < 0.001) and 11.0% (*P* = 0.002) of the variation, respectively. Log_10_(Lcn2) also had a significant effect on microbiota composition, accounting for 9.0% (*P* < 0.001) and 9.2% (*P* = 0.002) of the variation, using the same respective measures. Principal coordinates analysis (PCoA) of Bray-Curtis (Fig. 5A) and weighted UniFrac distances (Fig. S4G) also revealed a subset of *Hnf4a*^ΔIEC^ mice with distinct microbial community composition and high Lcn2 levels (Fig. 5B; S4H) compared to control mice. Using unweighted UniFrac distances that do not account for the relative abundance of microbial lineages, PERMANOVA revealed that genotype and log_10_(Lcn2) explained only 8.8% (*P* < 0.001) and 5.8% (*P* < 0.001) of the variation, which were less than the respective weighted UniFrac results. This suggests that the relative abundance of microbial taxa is an important contributor to the variation we observed between *Hnf4a*^ΔIEC^ and control mice. To determine which microbial taxa underlie the community differences, we first used DESeq2 to identify amplicon sequence variants (ASVs) that were significantly (*p*_adj_ <0.1) enriched or depleted in *Hnf4a*^ΔIEC^ mice compared to controls at 52 wk (Fig. 5C). This revealed 40 significantly differential ASVs in *Hnf4a*^ΔIEC^ mouse fecal microbiota compared to controls (Table S2; Fig. 5C). Analysis of the most abundant bacterial genera in these samples showed that *Hnf4a*^ΔIEC^ mice contained a relatively high abundance of *Akkermansia* (Fig. 5D; Fig. S4A and B). Closer inspection revealed that a single ASV identified as *Akkermansia muciniphila* (seq_4 in Table S2) had strikingly high relative abundance in *Hnf4a*^ΔIEC^ mice, although with a relatively modest fold enrichment in *Hnf4a*^ΔIEC^ compared to other enriched and depleted ASVs [DEseq2 log_2_(fold change) = −4.5, *p*_adj_ = 0.088] (Fig. 5E through J; Fig. S4E and F; Table S2). These results raised the possibility that *Akkermansia* abundance may be associated with inflammation in *Hnf4a*^ΔIEC^ mice. Indeed, log_10_(cumulative Lcn2) (Spearman *r* = 0.36) and pathology index (Spearman *r* = 0.35) were significantly correlated with *Akkermansia* relative abundance (Fig. S4C and D). Together these results indicate that *Hnf4a* deletion in the intestinal epithelium and associated inflammatory phenotypes are linked to alterations in microbiota composition at 1 yr of age and identify an association between *Akkermansia muciniphila* and the *Hnf4a*^ΔIEC^ mouse inflammatory phenotypes.

### *Akkermansia muciniphila* expansion in the gut microbiota of a subset of inflamed *Hnf4a*^ΔIEC^ mice early in life correlates with the onset of episodically elevated fecal Lcn2

**Fig 6:**
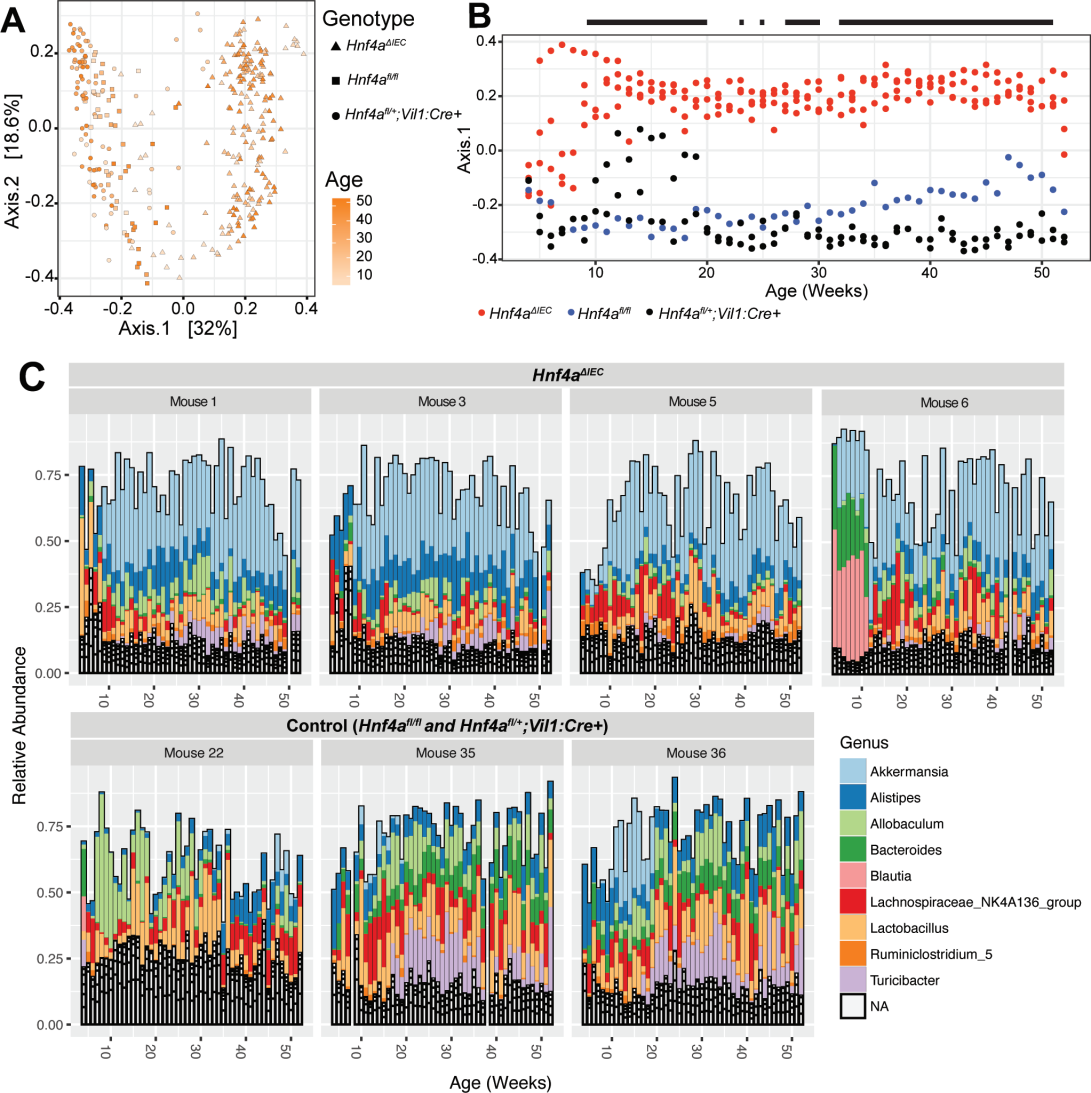
Longitudinal assessment of gut microbiota composition in a subset of inflamed *Hnf4a*^ΔIEC^ and control mice. (**A**) PCoA of Bray-Curtis dissimilarity colored by age. (**B**) Axis 1 of PCoA in (**A**) plotted against age in weeks and colored by genotype. The black bolded line above the plot represents timepoints at which genotype had a significant effect on Bray-Curtis dissimilarity. (**C**) Relative abundance of the top 20 most abundant genera in each longitudinal sample for each mouse. Genera labeled “NA” were not assigned taxonomic information at the genus level.

We next sought to understand the earlier stages of microbiota assembly that led up to the observed differences in a subset of inflamed *Hnf4a*^ΔIEC^ mice at 52 wk. We therefore performed a longitudinal profiling of 16S rRNA gene sequences of weekly fecal samples collected from *Hnf4a*^ΔIEC^ mice that displayed the highest lifetime cumulative Lcn2 through 52 wk of age (mice 1, 3, 5, and 6). We conducted the same analysis on three control mice (mice 22, 35, and 36) from the same experiment. PCoA of Bray-Curtis dissimilarity showed a clear separation between these selected *Hnf4a*^ΔIEC^ and control mice along axis 1 at the majority of timepoints (Fig. 6A). PERMANOVA of these longitudinal data confirmed that genotype, age, and genotype by age interaction had a significant effect on Bray-Curtis distances (all *P* < 0.001). To further resolve the life stages when those microbiota differences emerged, we plotted axis 1 of the PCoA (Fig. 6A) against age and then used a general linear model to test at which timepoints genotype had a significant effect on Bray-Curtis dissimilarity (Fig. 6B). The majority of timepoints after 9 wk were significant (*P* < 0.05). DEseq2 comparison of all *Hnf4a*^ΔIEC^ samples to all control samples in this longitudinal data set (Table S3) revealed enriched and depleted ASVs in this subset of mice. *Akkermansia* was the most abundant ASV across all samples and was significantly enriched in this subset of *Hnf4a*^ΔIEC^ mice compared to the selected controls. Other ASVs that were significantly enriched and depleted in this comparison are listed in Table S3. Relative abundance plots of the top 10 most abundant genera at each timepoint of this subset of mice clearly show a large relative increase in *Akkermansia* around 5–9 wk of age, which remained persistently high in this subset of *Hnf4a*^ΔIEC^ mice but not controls (Fig. 6C).

Since the *Hnf4a*^ΔIEC^ mice we included in this longitudinal analysis all showed histopathologic features of colitis by 52 wk, we tested if any microbial taxa were associated with episodically elevated fecal Lcn2 using two different methods. First, we used the ALDEx2 bioconductor package to test if any ASVs correlated with log_2_(Lcn2) values at matched timepoints in this subset of *Hnf4a*^ΔIEC^ mice (Table S4). Indeed, centered log-transformed counts of the most abundant *A. muciniphila* ASV (seq_1 in Tables S3 and S4) were significantly positively correlated with log_2_(Lcn2) (Table S4), along with 22 other ASVs, including those identified at the genus level, such as *Alistipes, Ruminiclostridium* 5*, Anaerofilum, Ruminococcaceae* UCG-014, and *Turicibacter*. ASVs identified as genus *Lachnospiraceae* UCG-006*, Lachnospiraceae* NK4A136 group*,* and of the family *Ruminococcaceae* were identified as significantly anti-correlated with log_2_(Lcn2). The second method we used tested if changes in Lcn2 from week to week correlate with changes in each ASV across the same timepoints across all seven selected mice. We found that only ASV_171, assigned to *Lachnospiraceae* NK4A136 group*,* was significant after multiple hypothesis testing correction. This suggests that a bacterial taxon belonging to *Lachnospiraceae* NK4A136 group is negatively correlated with episodically elevated fecal Lcn2 (Table S4). Together, the results of this longitudinal analysis in a subset of the most inflamed *Hnf4a*^ΔIEC^ mice reveal that their microbiota composition deviates from those of selected WT controls as early as 9 wk of age, including pronounced enrichment of *A. muciniphila*. However, this experimental design in which only a subset of animals were selected for longitudinal analysis limits our ability to make broad conclusions about the impact of host *Hnf4a* genotype on microbiota assembly.

### An *Akkermansia muciniphila* strain isolated from an aged, inflamed *Hnf4a*^ΔIEC^ mouse belongs to a new phylogroup of *Akkermansia muciniphila*

**Fig 7:**
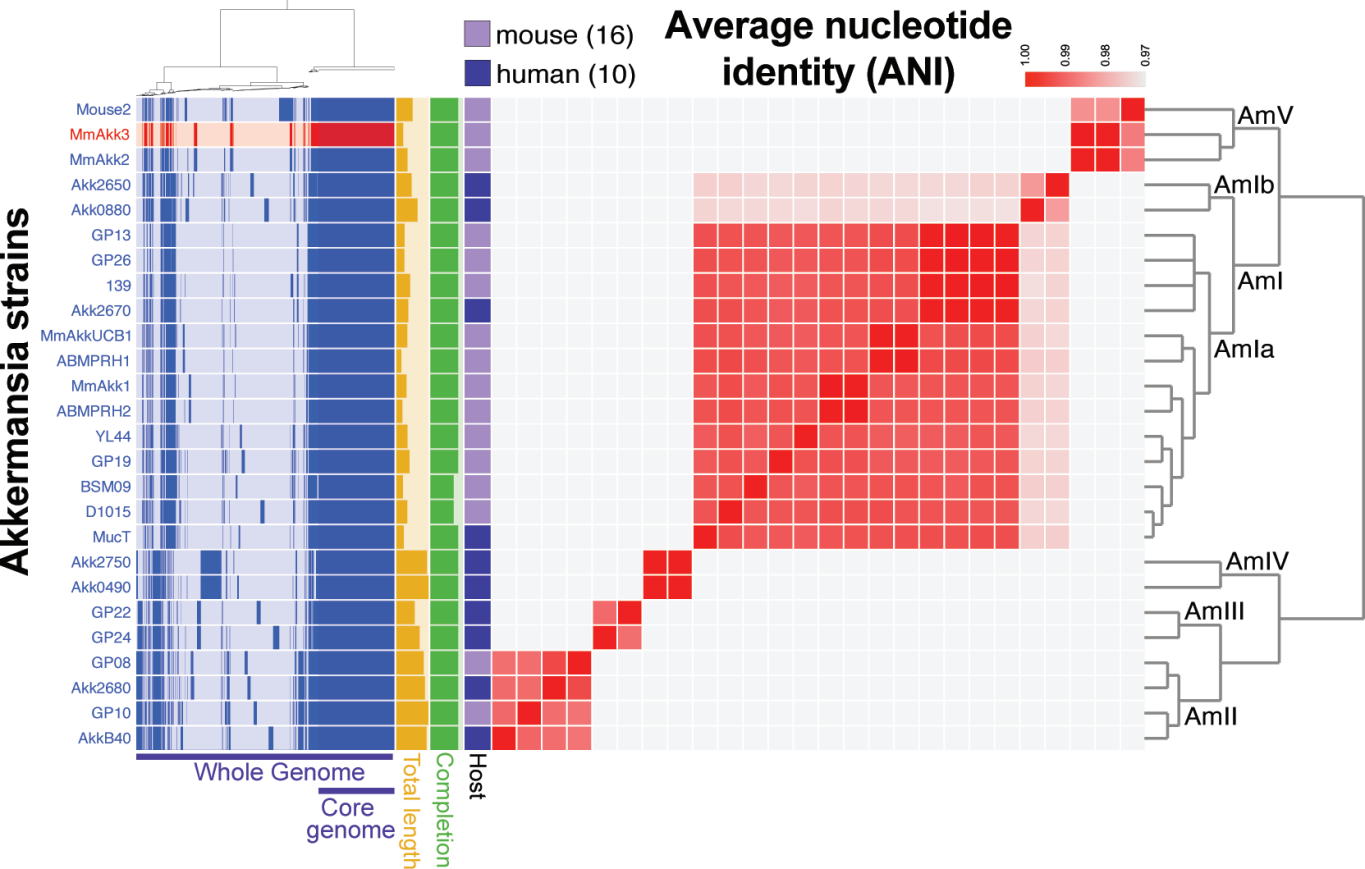
Pangenomic comparison of *A. muciniphila* strains. Comparison of human and mouse *A. muciniphila* isolate genomes using Anvi’o. The MmAkk3 strain isolated from *Hnf4a*^ΔIEC^ feces is highlighted in red. Genomes from previously published human isolates used as representative strains for each *Akkermansia* phylogroup are indicated in Table S5. Clustering in the phylogram (left) is based on gene frequency and displays the presence/absence of gene clusters for each genome. Anvi’o and PyANI were used to calculate the average nucleotide identity (ANI). Total genome length ranges between 2,500,000 and 3,208,715 bp. % Completion, which refers to how complete the assembled genome sequence is, ranges between 90% and 100%.

The striking enrichment of an *Akkermansia* ASV in *Hnf4a*^ΔIEC^ mice in our 16S rRNA gene sequencing data motivated us to characterize the specific *Akkermansia* strain underlying that enrichment. We cultured a fecal sample from an aged, inflamed *Hnf4a*^ΔIEC^ mouse on mucin medium and isolated a single strain that was identified as a variant of *Akkermansia muciniphila* by 16S rRNA gene sequencing (hereafter named MmAkk3). The 16S rRNA gene sequence from this strain perfectly matched the reference sequence of the single *Akkermansia* ASV we found to be enriched in our earlier microbiota analysis. To provide context for understanding this colitis-associated strain, we sequenced its genome and performed a comparative analysis with 26 other *A. muciniphila* genomes representing strains from various established *Akkermansia* phylogroups (55), including other mouse isolates (Table S5). While the majority of mouse isolates clustered together in the phylogroup AmIa, MmAkk3 was one of the three strains that clustered in a new phylogroup, which we named AmV. The other two strains in AmV included a strain we isolated at Duke University from a mouse lacking the major intestinal mucin *Muc2,* which displays chronic intestinal inflammation (43), and a strain available on NCBI, which was isolated from a wild-type mouse at Institute of Microbiology, ETH Zurich, Switzerland (Fig. 7; Table S5). Anvi’o functional enrichment analysis (56) revealed several potential functions, protein families, and pathways that were unique or enriched in AmV or enriched in phylogroups other than AmV (Fig. S5; Table S5). For example, immunoglobulin A1 protease and beta glucosidase were enriched in AmV, and mannosyltransferase and glycosyl hydrolase family 88 were significantly enriched in non-AmV groups (Table S5). Overall, these analyses outline genetic differences in a new phylogroup of *Akkermansia,* which contains two members isolated from mouse models of intestinal inflammation (*Hnf4a*^ΔIEC^ and *Muc2^−/−^*).

## DISCUSSION

Despite significant advances in clinical management of human IBD, the subclinical stages that lead to IBD remain poorly understood and difficult to study, yet represent unique windows for early diagnosis and therapy. A retrospective study found that IBD patients were more likely to report GI symptoms to their primary care provider in 5 yr before IBD diagnosis compared to the general population (57). First-degree relatives of CD patients also display subclinical intestinal inflammation, which can be detected by measuring fecal calprotectin, a biomarker used more commonly than Lcn2 in humans (58, 59). A long preclinical phase of asymptomatic mucosal inflammation in CD is supported by serologic markers evident 5 yr before diagnosis (60) and altered mucosal permeability in first-degree relatives of CD patients (61 - 63). In a clinical setting, the term “flare” is typically used to connote a clinical reactivation of disease. Our mouse data support the possibility that subclinical inflammation flares dynamically in humans who are predisposed to IBD and that these patterns could be detected by longitudinal fecal sampling and analysis. Future prospective cohort studies aimed at early IBD detection could include longitudinal assessments of fecal Lcn2, calprotectin, microbiota, or other subclinical biomarkers to non-invasively search for flaring patterns in relatives of IBD patients, patients with IBD risk alleles at the *HNF4A* locus, or patients with frequent GI symptoms that have not yet been diagnosed with IBD. Non-coding *HNF4A* variants have been associated with IBD (28, 37), but the impact of those variants on HNF4A expression remains unclear. Coding mutations in *HNF4A* have been linked to MODY1 (64, 65), but to our knowledge, MODY1 individuals have not been reported to have increased IBD risk. Therefore, the clinical relevance of this mouse model of IEC-specific *HNF4A* deletion may be limited but nonetheless provides an opportunity to investigate general principles of HNF4A-microbiota interaction during early subclinical stages of IBD. As early subclinical stages of IBD are better defined in humans, well-characterized animal models such as this can provide insight into the mechanisms underlying early subclinical IBD incidence and progression.

Our work here establishes the *Hnf4a*^ΔIEC^ mouse as a model for studying the progressive early subclinical stages of intestinal inflammation. We demonstrated the occurrence of episodically elevated Lcn2 and episodic loose stools in *Hnf4a*^ΔIEC^ mice throughout life under SPF conditions and found that cumulative lifetime Lcn2 correlates with endpoint histopathological index. We speculate that the *Hnf4a*^ΔIEC^ gut is more susceptible to sporadic microscopic injury and transient inflammatory events due to the role of HNF4A in intestinal processes, including barrier function (33), epithelial differentiation and absorptive function (30, 34, 35), colonic ion transport (31), and immune responses (36) including intra-epithelial lymphocyte-IEC interaction (32). Episodically elevated Lcn2 may signify short-lived inflammatory events in the *Hnf4a*^ΔIEC^ gut that are self-resolving but can become perpetuated in a subset of animals leading ultimately to the development of colitis. Weekly assessment of fecal Lcn2 was useful for this study, but that assay offers only a limited view of the dynamic and complex processes underlying inflammation and resolution. We note the penetrance and severity of inflammation we observed in SPF *Hnf4a*^ΔIEC^ mice are lower than previously reported in a separate SPF facility (31). These differences may be due to different environmental factors such as microbiota composition, diet, and housing, or subtle differences in genetic background (66). We also note that one particular cage in our SPF study housed the most inflamed mice (cage 1, mice 1–3) for unknown reasons. Within this experiment, diet and facility conditions were the same between cages, and there was no evidence of mouse aggression within the cage. Future studies in this animal model hold the potential to reveal mechanisms and additional molecular markers surrounding episodic inflammation that could be eventually applied to early subclinical and clinical stages of human IBD. Moreover, this *Hnf4a*^ΔIEC^ model could provide a sensitive background that could be used to identify dietary, microbial, genetic, or other factors that exacerbate or protect from early subclinical and subsequent clinical gut inflammation.

Our results reveal that microbiota colonization status and *Hnf4a*^ΔIEC^ genotype interactively influence intestinal phenotypes. Using antibiotic depletion and gnotobiotic approaches, we show that episodically elevated fecal Lcn2, episodic loose stools, and histopathological features of colitis are all microbiota dependent in this model. This is consistent with several other animal models of IBD, in which microbiota are required for induction or exacerbation of colitis or ileitis (4, 8 - 12 - 67) or associated gene expression patterns (41). We previously reported that GF mice displayed a genome-wide increase in HNF4A chromatin occupancy in intestinal epithelial cells compared to colonized controls (41). This raised the possibility that *Hnf4a*^ΔIEC^ mice reared in the absence of microbes, a state in which HNF4A activity is escalated in wild-type mice (41), may display a marked intestinal phenotype. However, we observed only minimal signs of inflammation and no other overt phenotypes in GF *Hnf4a*^ΔIEC^ mice compared to GF controls.

Our year-long longitudinal analyses in *Hnf4a*^ΔIEC^ and control mice provide an important overview of disease progression, and we explored microbiota composition in that context. A previous report in this model at 3 wk of age revealed no significant difference in microbiota composition between SPF *Hnf4a*^ΔIEC^ mice and controls (32). Our results extend this understanding by assessing microbiota composition at 52 wk of age, when some mice are inflamed. We also provide a description of longitudinal microbiota composition changes that occurred over 1 yr in a subset of the most inflamed *Hnf4a*^ΔIEC^ mice. Here, we only performed 16S rRNA gene profiling to define bacterial communities, though future studies could apply metagenomics or metabolomics to further resolve differences in microbiota composition and physiologic potential. Nevertheless, we identified several bacterial ASVs enriched and depleted in our *Hnf4a*^ΔIEC^ mice at 52 wk. Several ASVs belonging to *Clostridium sensu stricto 1* were among the top enriched ASVs in *Hnf4a*^ΔIEC^ mice, while an ASV belonging to the family *Lachnospiraceae* was the most depleted ASV in *Hnf4a*^ΔIEC^ mice. *Clostridium sensu stricto 1* has been positively associated with intestinal inflammation in a porcine model of IBD (68) and also in fecal samples of IBD patients (69). In contrast, it was significantly reduced in first-degree relatives of CD patients with impaired barrier function (70). *Lachnospiraceae* family members produce short-chain fatty acids that promote intestinal homeostasis (71) and have been shown to be reduced in treatment-naïve pediatric CD patients (72). In our 52-wk data set, we also observed that *A. muciniphila*, though it was only modestly enriched in *Hnf4a*^ΔIEC^ mice compared to controls according to DEseq2, had a very high relative abundance in several *Hnf4a*^ΔIEC^ mouse samples that correlated with both pathology index and log_10_(cumulative Lcn2). Notably, a salient increase in *A. muciniphila* relative abundance was observed by 9 wk of age in the subset of inflamed *Hnf4a*^ΔIEC^ mice that we longitudinally evaluated. *A. muciniphila* is a mucin-digesting member of the gut microbiota in humans, mice, and other animals (73). It has been associated with metabolic health in humans (74) and facilitates wound repair in the mouse colon (75). However, it also promotes susceptibility to intestinal pathogens in mice fed with a fiber-deficient diet (76) and colitis in mice with genetic susceptibility to IBD (77). *A. muciniphila* has also been shown to elicit antigen-specific T-cell-dependent IgG1 responses by the host under homeostatic conditions, setting it apart from most commensal microbes (78). Future characterization of the role of *Akkermansia* strains in this model using monoassociation studies could help uncover whether the inflamed *Hnf4a*^ΔIEC^ gut environment enriches for *Akkermansia* and whether *Akkermansia* promotes inflammation in *Hnf4a*^ΔIEC^ mice. Similar approaches could also be used to determine if other bacterial strains enriched in *Hnf4a*^ΔIEC^ mice are sufficient to drive inflammatory phenotypes. The spatial localization of *Akkermansia* and mucus layer organization were not evaluated in this study, but we did observe reduced proximal colon goblet cells and increased ileal goblet cells (Fig. 2K and 1C). Previous reports have shown significant increases in goblet cell number in the jejunum of *Hnf4a*^ΔIEC^ mice (79), which may be due to the role of HNF4 transcription factors in promoting enterocyte identity in the small intestine (53). Our comparative genomic analysis established that the *A. muciniphila* strain enriched in our *Hnf4a*^ΔIEC^ mice in this study is a founding member of a new phylogroup we refer to as AmV. As examination of the *Akkermansia* genus deepens (55, 80), the *Akkermansia* research community will need to work toward consensus on how new phylogroups and species may be defined. For now, we refer to the strains in AmV as members of a novel *A. muciniphila* phylogroup. Another founding member of AmV was cultivated from a knockout mouse IBD model lacking the major intestinal mucin Muc2 (*Muc2^−/−^*). Considering the goblet cell phenotypes, we report here in *Hnf4a*^ΔIEC^ mice, it will be interesting to determine if alteration of mucus production or other aspects of goblet cell physiology alters the selective landscape for different *Akkermansia* phylogroups or other mucus-digesting microbiota members.

## MATERIALS AND METHODS

### Animals

All mouse experiments were approved by Duke University’s Institutional Animal Care and Use Committee (protocol A125-20-06). *Hnf4a*^ΔIEC^ mice and littermate controls were generated using *Hnf4a*^fl/+^ (stock no. 004665) and *Vil1:Cre+* mice (stock no. 004586) from Jackson Laboratories (Bar Harbor, ME, USA). Upon purchase, *Hnf4a*^fl/+^ and *Vil1:Cre+* lines had been backcrossed to the C57BL/6J background 5 and 19 times, respectively, and we did not backcross further. *Muc2*^−/−^ mice (81) were gifted by Leonard Augenlicht (Albert Einstein College of Medicine, The Bronx, NY, USA). Mice were housed under SPF conditions (*Helicobacter, Pasteurella,* and murine norovirus free) on LabDiet 5053 diet and Enrich-o’Cobs bedding (The Andersons 4EB) at Duke University.

Pregnant female *Hnf4a*^fl/fl^ or *Hnf4a*^fl/+^;*Vil1:Cre+* mice were transferred from Duke to the National Gnotobiotic Rodent Resource Center (University of North Carolina-Chapel Hill, Chapel Hill, NC, USA) for cesarean section derivation into GF conditions. GF pups were fostered by Swiss webster dams, transferred to the Duke Gnotobiotic Core Facility at 8 wk of age, and then bred and housed in sterile Trexler isolators on Teklad Global Soy Protein-Free Extruded Rodent Diet (Sterilizable, Envigo 2020SX) and Alpha-dri (Shepherd) bedding for all gnotobiotic experiments.

### Additional methods

Additional methods for fecal Lcn2 enzyme-linked immunosorbent assay (ELISA), histology, statistical analysis, antibiotic treatment and gnotobiotic husbandry, 16S rRNA gene sequencing analysis, and *Akkermansia* culture and comparative genomic analysis are available in Text S1 in the supplemental material.

## Data Availability

Code used for 16S rRNA gene sequencing data analysis in this manuscript is available on GitHub. Sequencing reads generated as part of this study are available at Sequence Read Archive under BioProject ID PRJNA945427.

## SUPPLEMENTAL MATERIAL

Supplemental material mbio.01504-23-s0001.pdf Supplemental methods and Fig. S1 to S5.

Table S1 mbio.01504-23-s0002.docx *P*-values for gnotobiotic experiment.

Table S2 mbio.01504-23-s0003.xlsx Fifty-two-week 16S rRNA gene sequencing DEseq2 analysis and ASV table.

Table S3 mbio.01504-23-s0004.xlsx Longitudinal 16S rRNA gene sequencing DEseq2 analysis and ASV table.

Table S4 mbio.01504-23-s0005.xlsx Longitudinal 16S rRNA gene sequencing ASV counts vs log(Lcn2) ALDEx2 and Spearman correlation analysis results.

Table S5 mbio.01504-23-s0006.xlsx *Akkermansia* strain list, heatmap values for Figure S5, and summary of significantly enriched COGs, pfams, KEGG pathways in AmV or non-AmV *Akkermansia* strains.

ASM does not own the copyrights to Supplemental Material that may be linked to, or accessed through, an article. The authors have granted ASM a non-exclusive, world-wide license to publish the Supplemental Material files. Please contact the corresponding author directly for reuse.
