# Supplementary material for: Spontaneous episodic inflammation in the intestines of mice lacking HNF4A is driven by microbiota and associated with early life microbiota alterations
Source: mBio. 2023 Aug 1;14(4):e01504-23. doi: 10.1128/mbio.01504-23 (PMC10470520; doi:10.1128/mbio.01504-23)
Supplement: Supplemental material — Supplemental methods and Fig. S1 to S5. [file mbio.01504-23-s0001.pdf]

Spontaneous episodic inflammation in the intestines of mice lacking HNF4A is driven by microbiota and associated with early-life microbiota alterations

Cecelia Kelly[1], Jayanth Jawahar[1], Lauren Davey[1,2], Jeffrey I. Everitt[3], Joseph A. Galanko[4], Chelsea Anderson[4], Jonathan E. Avendano[1], Jessica R. McCann[1], R. Balfour Sartor[4], Raphael H. Valdivia[1,2], and John F. Rawls[1]

[1]Department of Molecular Genetics and Microbiology, Duke Microbiome Center, Duke University School of Medicine, Durham, NC 27710, USA.
[2]Department of Immunology, Duke University School of Medicine, Durham, NC 27710, USA
[3]Research Animal Pathology Core, Department of Pathology, Duke University School of Medicine, Durham, NC 27710, USA.
[4]Center for Gastrointestinal Biology and Disease, Department of Medicine, University of North Carolina at Chapel Hill, Chapel Hill, NC 27599, USA.

Correspondence to john.rawls@duke.edu

SUPPLEMENTAL MATERIALS

Text S1: Supplemental methods

Fecal sample prep and Lcn2 ELISA

Fresh fecal samples were collected from individual mice into sterile tubes and stored at -80° C. Samples were prepared as previously described (1), and diluted 1:150, 1:9000, or 1:180000 for ELISA. DuoSet murine Lcn2 ELISA kit (R&D DY1857) was used, following kit instructions. Plates were read using a BioTek® Synergy H1 hybrid plate reader.

Tissue sampling and Histology

Following CO_2 euthanasia, small and large intestine was dissected out, and attached fat was trimmed away. Then the small intestine was further divided into duodenum (proximal quarter), jejunum (next 2 quarters), and ileum (most distal quarter). Intestinal segments were swiss rolled (2) and fixed in zinc buffered formalin (Thermo Scientific 5701ZF) at room temperature overnight, then processed, sectioned and stained with haemotoxylin and eosin (H&E) by the Duke Pathology Research Immunohistology Lab.

Histological image analysis and scoring

Histological scores of H&E stained colon sections were assigned by a board-certified veterinary pathologist in a masked fashion without knowledge of allocation group. Due to the mild, segmental, and variable phenotype, germ-free control animals were used as a baseline reference control group, and a scoring system to subjectively and semi-quantitatively assess inflammation was developed based on lamina propria inflammatory infiltrates and increased crypt hyperplasia in the proximal, middle, and distal colon. Each mouse received a score of 0=no change, 1=minimal, 2=mild, or 3=moderate or 4=severe for both lamina propria inflammatory infiltrates (inflammation score) and crypt hyperplasia for each segment of the colon (proximal, middle and distal). The sums of the scores for each segment were taken as the total colon inflammation and total colon crypt hyperplasia scores for each mouse. The sum of

total colon inflammation and total colon crypt hyperplasia scores was taken as the pathology index for each mouse.

40x scans of H&E stained slides were made with a Leica Aperio GT450 slide scanner by Duke Pathology Research Immunohistology Lab. Quantitative measurements of crypt length and goblet cell number were made by CK in a blinded fashion. Crypt length was measured with ObjectiveView software. Whole crypts with good cross-sectioning were selected for quantification. Goblet cells were counted by eye. 12 crypts were quantified per proximal (innermost loops until rugae end), middle (loops in between proximal and distal loops), and distal colon (outermost ~2 loops) segment, then averaged within their colonic segment to generate a single average measurement per colon segment per mouse.

Statistical analysis

Longitudinal repeated measures data in Figures 1, 3, and 4 were analyzed as follows: each outcome of interest (weight, episodic loose stools and lipocalin) was examined with a repeated measures regression. In the antibiotic treatment experiment, % of starting body weight was examined instead of raw body weight. In the GF/CV experiment, only lipocalin was examined. Weight and lipocalin were continuous variables. Episodic loose stools was dichotomized into none vs mild/severe. The predictors were genotype, sex, age in weeks, genotype by age in weeks, and genotype by sex interaction for the SPF experiment. For the antibiotic treatment experiment, the predictors were treatment, sex, timepoint and sex by treatment interaction. For the GF/CV experiment, the predictors were genotype, colonization status, life stage, and all the two-way and three-way interactions of these three predictors. For weight and lipocalin, a three-level genotype variable was used. For loose stools, wildtype and heterozygous were combined and a two-level genotype variable. Terms for the experiment number (SPF experiments only) and the cage number within experiment were included in the models. An auto-regressive correlation matrix was used to account for the repeated measures across mice over time. Analyses were performed using SAS Version 9.4 (SAS Institute, Cary, NC). P-values less than 0.05 were considered statistically significant.

To test the hypothesis that instances of Lcn2 >300 ng/g and episodic loose stools were coincident on the same day more often than expected by chance, we calculated the chance that *Hnf4a*$^{\Delta IEC}$ mice would have Lcn2 >300 ng/g at any given timepoint (161/1019 = 0.158). We also calculated the chance that *Hnf4a*$^{\Delta IEC}$ mice would have an episode of loose stools at any given timepoint (229/1019 = 0.2247). Then we multiplied these to probabilities to calculate the expected chance of these two phenomena being coincident if they are independent (0.158*0.2247 = 0.0355). We used a Chi squared test to test whether the actual distribution of timepoints with only Lcn2 >300 ng/g, only loose stools, both, or neither was significantly different from the expected distribution.

All p-value calculations, excluding longitudinal repeated measures analyses, were performed using PRISM 9 using the tests indicated in the figure or figure legend.

Antibiotic treatment to deplete microbiota

Adult SPF *Hnf4a*$^{\Delta IEC}$ mice were randomly assigned to treatment and control groups. Then, at three timepoints prior to treatment, fecal samples were taken for later assessment of pre-treatment Lcn2 levels. Treatment group mice were then treated continuously with 1 g/L ampicillin in the drinking water, and orally gavaged every 12 hours with a solution of 50 mg/kg body weight vancomycin, 100 mg/kg neomycin, 100 mg/kg metronidazole, and 0.5 mg/kg fluconazole for two weeks. Control mice were mock gavaged with reverse osmosis (RO) water.

To determine the efficacy of antibiotic treatment, fecal samples from selected timepoints were diluted to 50 mg/mL and DNA was extracted using the Quick-DNA Fecal/Soil Microbe miniprep kit (Zymo Research #D6010) according to manufacturer’s instructions. qPCR was

performed on extracted DNA using universal 16S rRNA gene primers 357F (forward) CTCCTACGGGAGGCAGCAG (3) and CD (reverse) CTTGTGCGGGCCCCCGTCAATTC (4).

Conventionalization of GF mice

Fecal samples collected from SPF *Hnf4a*$^{fl/+}$;*Vil1:Cre*+ and *Hnf4a*$^{fl/fl}$ mice were homogenized in an anaerobic chamber (Coy Laboratory, 5% hydrogen, 5% carbon dioxide, and 90% nitrogen) in PBS + 0.05% cysteine + 10% glycerol to make a 10% w/v fecal slurry. The slurry was centrifuged for 2 minutes at 300xg to pellet large particles, and aliquoted into sterile Eppendorf tubes and frozen at -80 C. CV-to-be mice were gavaged with 120 uL of thawed slurry. GF mice were not gavaged.

16S rRNA gene amplification and sequencing

DNA was extracted from mouse fecal pellets using the Quick-DNA Fecal/Soil Microbe miniprep kit (Zymo Research #D6010) according to manufacturer's instructions, then used for either 16S rRNA sequencing or qPCR. The Duke Microbiome Shared Resource (MSR) determined sample DNA concentration with a Qubit dsDNA HS assay kit (ThermoFisher, Q32854). Then the V4 variable region of the 16S rRNA gene was amplified by polymerase chain reaction using the forward primer 515 and reverse primer 806 following the Earth Microbiome Project protocol (http://www.earthmicrobiome.org/), which carry unique barcodes allowing for multiplexed sequencing. Equimolar 16S rRNA gene PCR products from all samples were quantified and pooled. Sequencing was then performed by the Duke Sequencing and Genomic Technologies shared resource on an Illumina MiSeq instrument configured for 250 base-pair paired-end SP sequencing run. Reads were converted to fastq files and demultiplexed by MSR.

Statistical analysis of 16S rRNA gene sequencing data

Raw data from 16S rRNA gene sequencing was processed using the DADA2 v1.8 package in the R statistical programming environment v3.4.0 (5, 6). In brief, raw demultiplexed reads were filtered using the *fastqPairedFilter* function, with *maxEE* set to 2, *rm.phix* set to TRUE to remove reads that match against the phIX genome, and other settings set to their defaults. Paired-end reads were merged using the *mergePairs* command, after which chimeric sequences were removed using the *removeBimeraDenovo* command using the method "consensus". Following chimera removal, taxonomy was assigned using Silva v123, with exact species matches assigned using the *addSpecies* function (7). The phyloseq package was used to combine the resulting data into a phyloseq object for subsequent analysis. All statistical analyses and graphical representations of the output phyloseq object were conducted in the R statistical programming environment using the phyloseq, vegan, and ggplot packages (8-10). Code used for 16S rRNA gene sequencing data analysis in this manuscript is available at: https://github.com/jawah003/hnf4a-mice-manuscript-2022. Sequencing reads generated as part of this study are available at sequence read archive under bioproject ID: PRJNA945427.

Isolation and culture of *A. muciniphila* strains from mouse fecal samples

All *A. muciniphila* isolation and culture procedures were performed under anaerobic conditions (Coy Laboratory anaerobic chamber, 5% hydrogen, 5% carbon dioxide, and 90% nitrogen). Approximately 10 mg of frozen fecal pellet (half pellet) was used to inoculate 5 ml of mucin medium and incubated at 37°C for 48 h. After three sequential passages in mucin medium, a sample of the suspension was streaked on 1% agar BBL brain heart infusion (BHI, BD Biosciences; catalog 211065) plates supplemented with 0.2% mucin and incubated for 6-7 days at 37°C. Colonies were purified by restreaking on fresh BHI mucin plates and incubated for 4 days. Pure isolates were grown in mucin medium for 5 days and frozen at -80 C in 25%

glycerol for storage. Total DNA was isolated, and the strains were identified by PCR-based amplification of the 16S rRNA gene using 27F (5' AGA GTT TGA TCC TGG CTC AG) and 1492R (5' GGT TAC CTT GTT ACG ACT T) primers.

Whole genome sequencing and assembly for isolated *A. muciniphila* strains

Strains from glycerol stocks were inoculated into synthetic media (11) and grown to saturation and pelleted. DNA was extracted from bacterial pellets using the Magattract® high molecular weight (HMW) DNA kit (QIAGEN 67563) according to manufacturer instructions for Gram-negative bacteria with the following modifications: 1) the proteinase K incubation step was extended to 1 hour, and 2) for steps involving incubation on a mixer, a vortex was used at max speed instead of a mixer. The extracted DNA was confirmed to be *A. muciniphila* by PCR with universal *Akkermansia* primers Muc-1129 and Muc-1437 (12). If necessary, extracted DNA was concentrated by ethanol precipitation.

Library preparation using the resulting DNA, sequencing, demultiplexing samples, conversion of resulting BAM files to fasta files, genome assembly, and subsequent annotation and quality evaluation of genomes was performed as written in (13). Genome assembly was performed with Flye version 2.7.1.

Comparative genomics of *A. muciniphila* strains

All *A. muciniphila* genome assemblies listed in NCBI Genome with either *mus musculus* listed as the host species or with an associated publication confirming that the strain originated from mice were selected for the pangenomic analysis (as of June 30, 2022; www.ncbi.nlm.nih.gov/genome/browse/#!/prokaryotes/1598/). Two assemblies in NCBI Genome were selected based on the paper describing their isolation from mice (14), although host information was not included in the BioSample metadata.

Comparative analysis of the assembled genomes was run using the pangenomic workflow in Anvi'o version 7.1 (15, 16). Anvi'o compatible contig databases were prepared with the script anvi-gen-contigs-database. This command uses Prodigal to identify open reading frames (17). The resulting databases were annotated with COGs, Pfam, and KEGG Orthologs (KOfam) using the following scripts: anvi-run-ncbi-cogs, anvi-run-pfams, and anvi-run-kegg-kofams. The genomes were subsequently combined to make a genome database using anvi-gen-genomes-storage. To compute the pangenome, the command anvi-pan-genome was run with the following parameters: --minbit 0.5, --mcl-inflation 10, --use-ncbi-blast.

The genome database was used to compute average nucleotide identity (ANI) across the genomes using the command anvi-compute-genome-similarity with the –method pyani parameter (18). To determine which phylogroup our isolates belonged to, we included publicly available *A. muciniphila* genomes with previously published phylogroup assignments as a control in our pangenome and ANI analyses. Strains representing phylogroups AmI, AmII, and AmIV isolated from human donors were described in Becken et al. (13). Strains belonging to phylogroup AmIII were described in Guo et al. (19). All representative genome assemblies were retrieved from NCBI (see Table S5 for accession information). Based on the resulting analysis, phylogroup specific gene functions and metabolic module enrichment were obtained using the commands anvi-compute-functional-enrichment-in-pan and anvi-compute-metabolic-enrichment.

Gene functions unique to AmV, missing from AmV but present in all other phylogroups, or present in AmI (the phylogroup with the majority of mouse strains) but absent in AmV were manually curated into Table S5. Metabolic pathways completeness analysis was determined using the command anvi-estimate-metabolism and heatmap visualization was performed using the R package pheatmap.

SUPPLEMENTAL METHODS REFERENCES

1. Oka A, Mishima Y, Liu B, Herzog JW, Steinbach EC, Kobayashi T, Plevy SE, Sartor RB. 2019. Phosphoinositide 3-Kinase P110delta-Signaling Is Critical for Microbiota-Activated IL-10 Production by B Cells that Regulate Intestinal Inflammation. Cells 8.
2. Bialkowska AB, Ghaleb AM, Nandan MO, Yang VW. 2016. Improved Swiss-rolling Technique for Intestinal Tissue Preparation for Immunohistochemical and Immunofluorescent Analyses. J Vis Exp doi:10.3791/54161.
3. Turner S, Pryer KM, Miao VP, Palmer JD. 1999. Investigating deep phylogenetic relationships among cyanobacteria and plastids by small subunit rRNA sequence analysis. J Eukaryot Microbiol 46:327-38.
4. Rudi K, Skulberg OM, Larsen F, Jakobsen KS. 1997. Strain characterization and classification of oxyphotobacteria in clone cultures on the basis of 16S rRNA sequences from the variable regions V6, V7, and V8. Appl Environ Microbiol 63:2593-9.
5. Callahan BJ, McMurdie PJ, Rosen MJ, Han AW, Johnson AJ, Holmes SP. 2016. DADA2: High-resolution sample inference from Illumina amplicon data. Nat Methods 13:581-3.
6. R Development Core Team. 2011. R: A language and environment for statistical computing, R Foundation for Statistical Computing, Vienna, Austria.
7. Quast C, Pruesse E, Yilmaz P, Gerken J, Schweer T, Yarza P, Peplies J, Glockner FO. 2013. The SILVA ribosomal RNA gene database project: improved data processing and web-based tools. Nucleic Acids Res 41:D590-6.
8. Oksanen J, Kindt, Roeland, Legendre, Pierre, O'Hara, Bob, Simpson, Gavin L., Solymos, Peter, Stevens, M. Henry H., Wagner, Helene. 2008. The vegan package, vol 10, p 631-637. Community Ecology Package.
9. McMurdie PJ, Holmes S. 2013. phyloseq: an R package for reproducible interactive analysis and graphics of microbiome census data. PLoS One 8:e61217.
10. Wickham H. 2016. ggplot2: elegant graphics for data analysis. Springer.
11. Plovier H, Everard A, Druart C, Depommier C, Van Hul M, Geurts L, Chilloux J, Ottman N, Duparc T, Lichtenstein L, Myridakis A, Delzenne NM, Klievink J, Bhattacharjee A, van der Ark KC, Aalvink S, Martinez LO, Dumas ME, Maiter D, Loumaye A, Hermans MP, Thissen JP, Belzer C, de Vos WM, Cani PD. 2017. A purified membrane protein from Akkermansia muciniphila or the pasteurized bacterium improves metabolism in obese and diabetic mice. Nat Med 23:107-113.
12. Collado MC, Derrien M, Isolauri E, de Vos WM, Salminen S. 2007. Intestinal integrity and Akkermansia muciniphila, a mucin-degrading member of the intestinal microbiota present in infants, adults, and the elderly. Appl Environ Microbiol 73:7767-70.
13. Becken B, Davey L, Middleton DR, Mueller KD, Sharma A, Holmes ZC, Dallow E, Remick B, Barton GM, David LA, McCann JR, Armstrong SC, Malkus P, Valdivia RH. 2021. Genotypic and Phenotypic Diversity among Human Isolates of Akkermansia muciniphila. mBio 12.
14. Geerlings SY, Ouwerkerk JP, Koehorst JJ, Ritari J, Aalvink S, Stecher B, Schaap PJ, Paulin L, de Vos WM, Belzer C. 2021. Genomic convergence between Akkermansia muciniphila in different mammalian hosts. BMC Microbiol 21:298.
15. Eren AM, Esen OC, Quince C, Vineis JH, Morrison HG, Sogin ML, Delmont TO. 2015. Anvi'o: an advanced analysis and visualization platform for 'omics data. PeerJ 3:e1319.
16. Delmont TO, Eren AM. 2018. Linking pangenomes and metagenomes: the Prochlorococcus metapangenome. PeerJ 6:e4320.
17. Hyatt D, Chen GL, Locascio PF, Land ML, Larimer FW, Hauser LJ. 2010. Prodigal: prokaryotic gene recognition and translation initiation site identification. BMC Bioinformatics 11:119.

18. Pritchard L, Glover RH, Humphris S, Elphinstone JG, Toth IK. 2016. Genomics and taxonomy in diagnostics for food security: soft-rotting enterobacterial plant pathogens. Analytical Methods 8:12-24.
19. Guo X, Li S, Zhang J, Wu F, Li X, Wu D, Zhang M, Ou Z, Jie Z, Yan Q, Li P, Yi J, Peng Y. 2017. Genome sequencing of 39 Akkermansia muciniphila isolates reveals its population structure, genomic and functional diverisity, and global distribution in mammalian gut microbiotas. BMC Genomics 18:800.

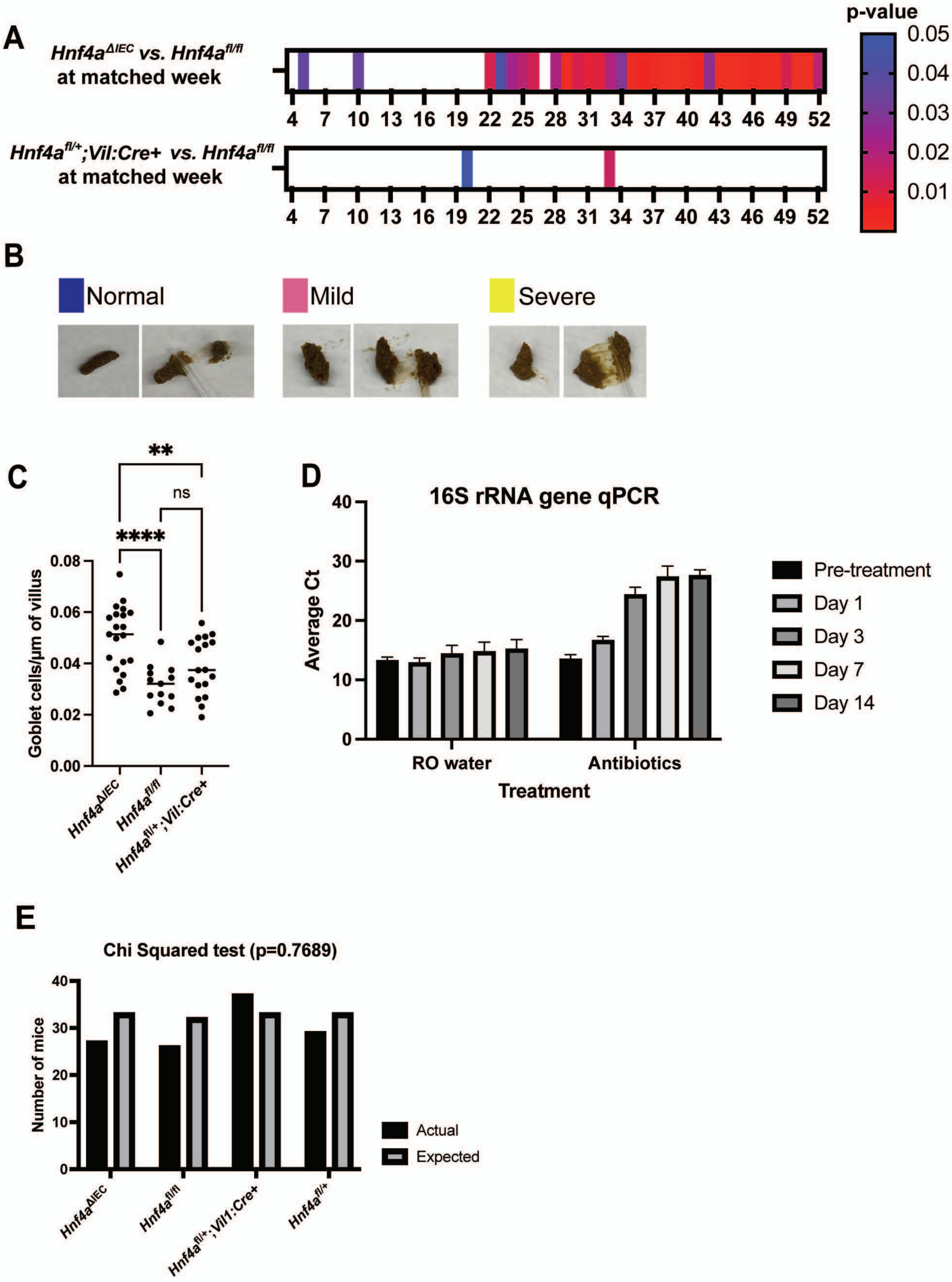
A
Hnf4aΔIEC vs. Hnf4afl/fl
at matched week
4 7 10 13 16 19 22 25 28 31 34 37 40 43 46 49 52
Hnf4afl/+;Vil:Cre+ vs. Hnf4afl/fl
at matched week
4 7 10 13 16 19 22 25 28 31 34 37 40 43 46 49 52
p-value
0.05
0.04
0.03
0.02
0.01
B
Normal
Mild
Severe
C
**
ns
****
Goblet cells/µm of villus
0.08
0.06
0.04
0.02
0.00
Hnf4aΔIEC
Hnf4afl/fl
Hnf4afl/+;Vil:Cre+
D
16S rRNA gene qPCR
Average Ct
40
30
20
10
0
RO water
Antibiotics
Treatment
Pre-treatment
Day 1
Day 3
Day 7
Day 14
E
Chi Squared test (p=0.7689)
Number of mice
40
30
20
10
0
Actual
Expected
Hnf4aΔIEC
Hnf4afl/fl
Hnf4afl/+;Vil1:Cre+
Hnf4afl/+

Figure S1: Supplemental data related to Figures 1-4.
(A) Heatmap showing the repeated measures regression p-value for the genotype by age interaction terms at matched weeks for the indicated genotype comparisons. Age in weeks is indicated along the x-axis. Non-significant p-values are colored white. (B) Representative images of 3-level scoring system we used for episodic loose stool severity. (C) Quantification of goblet cells in the ileum of SPF *Hnf4a*$^{\Delta IEC}$ and control mice (same mice as described in Figure 1). (D) Average Ct values for 16S qPCR on DNA extracted from mouse fecal samples at indicated timepoints throughout the antibiotic treatment. (E) Chi squared test comparing actual vs expected Mendelian distribution in GF mouse litters. P-values for (C) were calculated using an ordinary one-way ANOVA followed by Tukey's multiple comparison testing. **** p<0.0001, ** p<0.01, ns = not significant.

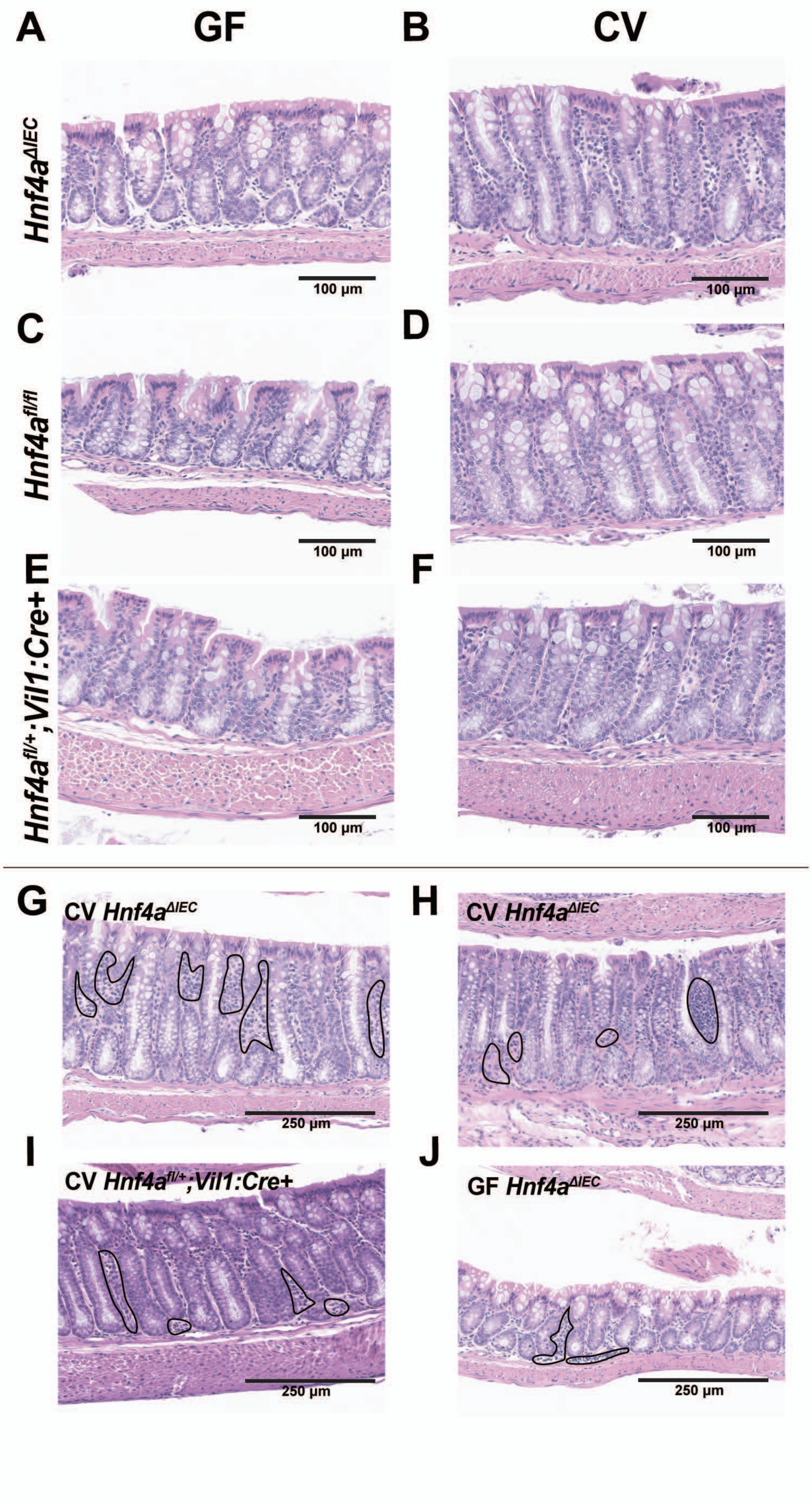

A
GF
B
CV
$Hnf4a^{ΔIEC}$
100 µm
100 µm
C
D
$Hnf4a^{fl/fl}$
100 µm
100 µm
E
F
$Hnf4a^{fl/+};Vil1:Cre+$
100 µm
100 µm
G
CV $Hnf4a^{ΔIEC}$
250 µm
H
CV $Hnf4a^{ΔIEC}$
250 µm
I
CV $Hnf4a^{fl/+};Vil1:Cre+$
250 µm
J
GF $Hnf4a^{ΔIEC}$
250 µm

Figure S2: Representative images of average crypt length and inflammatory infiltrates in different groups of gnotobiotic mice.
(A-F) Representative images of crypt length selected on the basis that their measurements fell near the mean of their respective group. (G-J) Example images showing inflammatory infiltrates selected by veterinary pathologist J.I. Everitt. We chose to show focal sites of inflammatory infiltrates in CV *Hnf4a*$^{\Delta IEC}$ mice, in which they were seen most frequently, but also CV and GF control mice to provide examples of what we saw in mice that received non-zero scores in all groups. Mouse colonization status and genotype are labeled, and areas of inflammatory infiltrates are outlined.

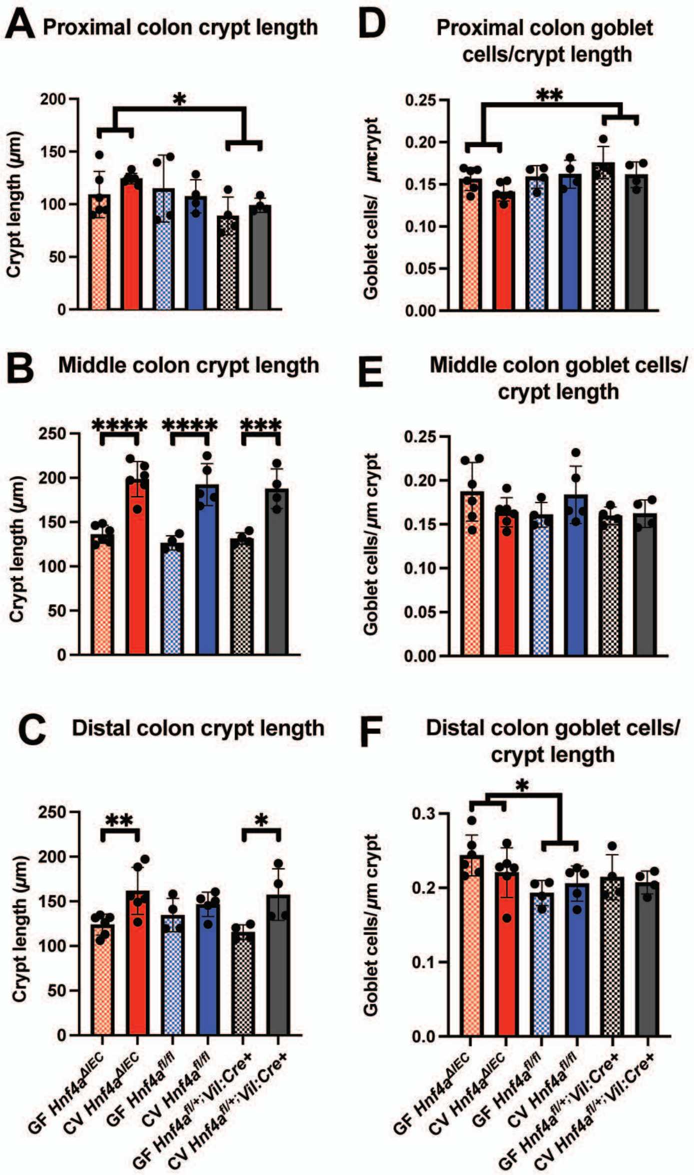
A
Proximal colon crypt length
Crypt length (µm)
200
150
100
50
0
*
D
Proximal colon goblet cells/crypt length
Goblet cells/ µm crypt
0.25
0.20
0.15
0.10
0.05
0.00
**
B
Middle colon crypt length
Crypt length (µm)
250
200
150
100
50
0
****
****
***
E
Middle colon goblet cells/ crypt length
Goblet cells/µm crypt
0.25
0.20
0.15
0.10
0.05
0.00
C
Distal colon crypt length
Crypt length (µm)
250
200
150
100
50
0
**
*
F
Distal colon goblet cells/ crypt length
Goblet cells/µm crypt
0.3
0.2
0.1
0.0
*
GF Hnf4aΔIEC
CV Hnf4aΔIEC
GF Hnf4afl/fl
CV Hnf4afl/fl
GF Hnf4afl/+;Vil:Cre+
CV Hnf4afl/+;Vil:Cre+

Figure S3: Quantification of crypt length and goblet cell number in GF and CV *Hnf4a*$^{\Delta IEC}$ and control mice.

(A-C) Average crypt length (μm) and (D-F) goblet cells/μm of crypt. A 2-way ANOVA was performed to test whether genotype, colonization status or genotype by colonization status interaction had a significant effect on either crypt length or goblet cell number. Genotype by colonization status interaction had no significant effect in any of the comparisons. However, when either genotype or colonization status was a significant factor in a comparison, either Sidak's or Tukey's multiple comparisons tests were performed. Those p-values are indicated in the figure panels using brackets. **** p<0.0001, ** p<0.01, * p<0.05.

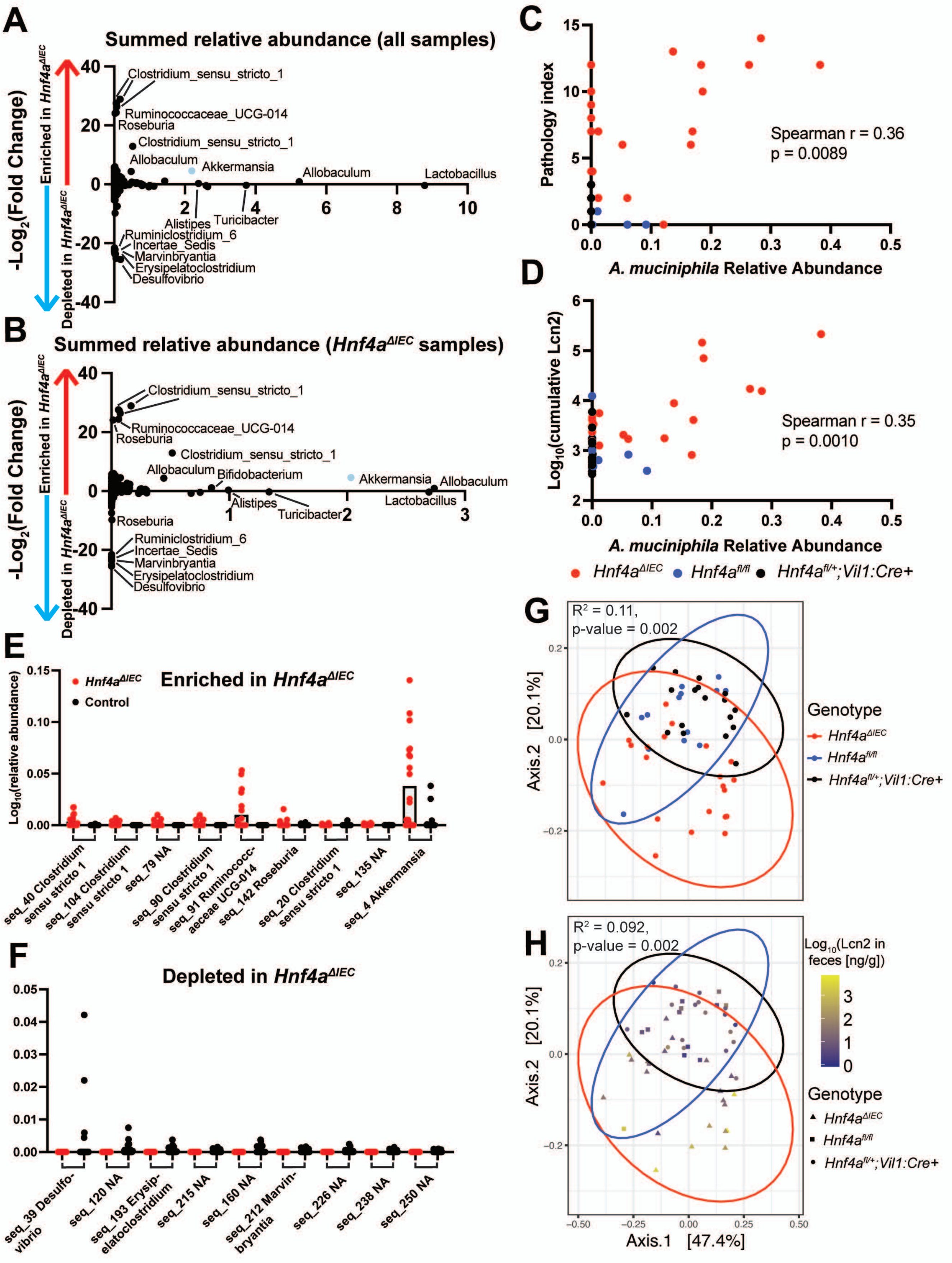

A
Summed relative abundance (all samples)
-Log2(Fold Change)
Enriched in Hnf4a^ΔIEC
Depleted in Hnf4a^ΔIEC
Clostridium_sensu_stricto_1
Ruminococcaceae_UCG-014
Roseburia
Clostridium_sensu_stricto_1
Allobaculum
Akkermansia
Allobaculum
Lactobacillus
Alistipes
Turicibacter
Ruminiclostridium_6
Incertae_Sedis
Marvinbryantia
Erysipelatoclostridium
Desulfovibrio
B
Summed relative abundance (Hnf4a^ΔIEC samples)
Bifidobacterium
C
Pathology index
A. muciniphila Relative Abundance
Spearman r = 0.36
p = 0.0089
D
Log10(cumulative Lcn2)
Spearman r = 0.35
p = 0.0010
Hnf4a^ΔIEC
Hnf4a^fl/fl
Hnf4a^fl/+;Vil1:Cre+
E
Enriched in Hnf4a^ΔIEC
Log10(relative abundance)
Control
seq_40 Clostridium sensu stricto 1
seq_104 Clostridium sensu stricto 1
seq_79 NA
seq_90 Clostridium sensu stricto 1
seq_91 Ruminococcaceae UCG-014
seq_142 Roseburia
seq_20 Clostridium sensu stricto 1
seq_135 NA
seq_4 Akkermansia
F
Depleted in Hnf4a^ΔIEC
seq_39 Desulfovibrio
seq_120 NA
seq_193 Erysipelatoclostridium
seq_215 NA
seq_160 NA
seq_212 Marvinbryantia
seq_226 NA
seq_238 NA
seq_250 NA
G
R² = 0.11,
p-value = 0.002
Axis.2 [20.1%]
Genotype
H
R² = 0.092,
p-value = 0.002
Log10(Lcn2 in feces [ng/g])
Axis.1 [47.4%]

Figure S4: Enrichment of *A. muciniphila* and other ASVs in *Hnf4a*$^{\Delta IEC}$ and control mice at 52 weeks of age.

(A-B) Summed relative abundance for each ASV across all samples (A) or only *Hnf4a*$^{\Delta IEC}$ samples (B) is plotted on the x-axis against -Log_2(Fold Change) on the y-axis to show where *A. muciniphila* falls compared to other ASVs along these two parameters. *A. muciniphila* is highlighted in light blue. (C-D) Spearman correlation of the indicated measures. (E-F) Plots of the Log_{10}(relative abundance) of the top 8 most enriched ASVs and seq_4 (*A. muciniphila*) (E), as well as top 9 most depleted ASVs (F) in *Hnf4a*$^{\Delta IEC}$ mice in endpoint 16S rRNA gene sequencing dataset determined by DEseq2 analysis. (G-H) PCoA of weighted UniFrac distance colored by (G) genotype and (H) Log_{10}(Lcn2) levels. 95% confidence ellipses are color coded to genotype. PERMANOVA R^2 and p values are indicated at the top of each figure panel, showing the percentage of variance explained by genotype and Log_{10}(Lcn2), respectively.

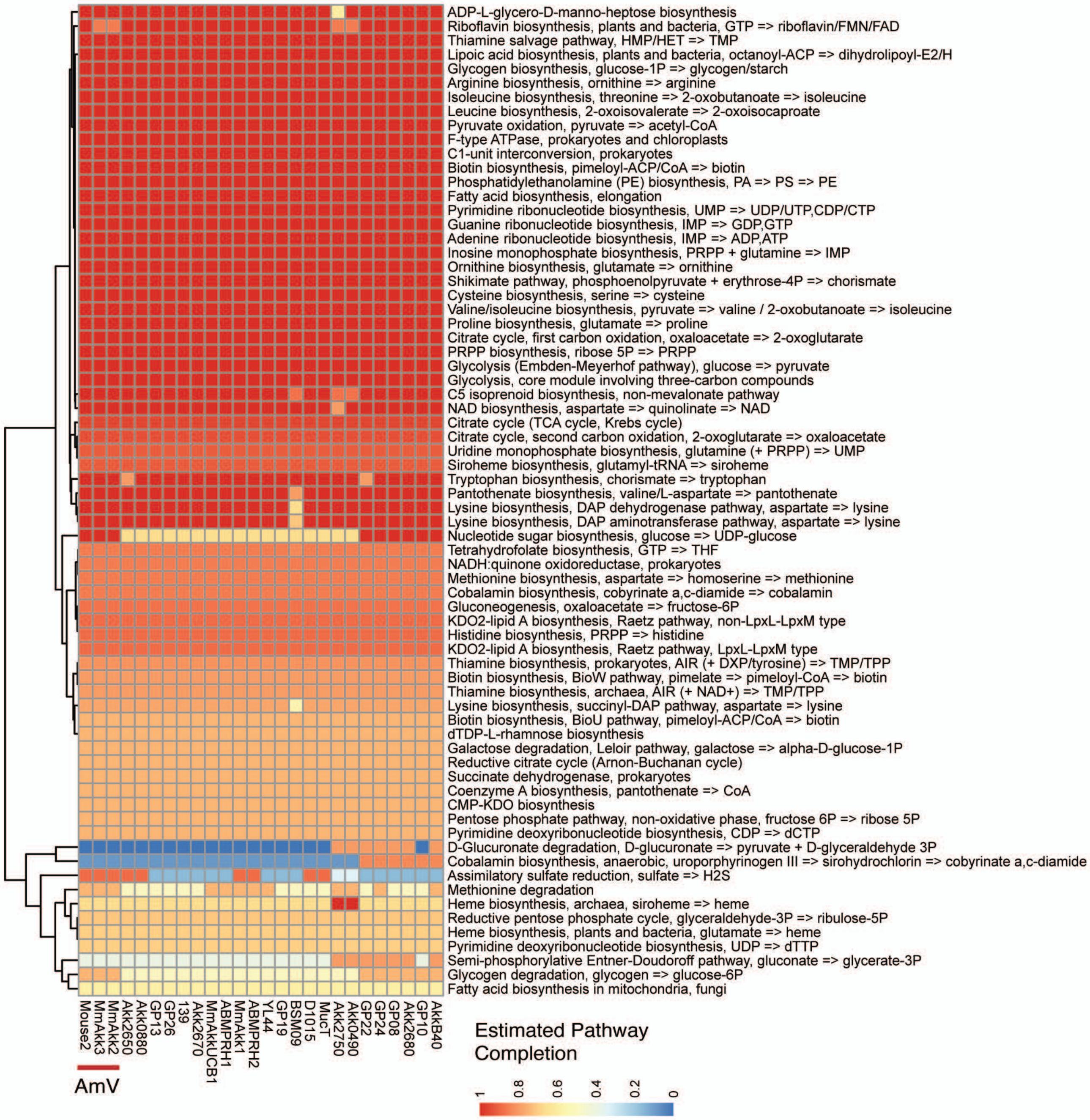
ADP-L-glycero-D-manno-heptose biosynthesis
Riboflavin biosynthesis, plants and bacteria, GTP => riboflavin/FMN/FAD
Thiamine salvage pathway, HMP/HET => TMP
Lipoic acid biosynthesis, plants and bacteria, octanoyl-ACP => dihydrolipoyl-E2/H
Glycogen biosynthesis, glucose-1P => glycogen/starch
Arginine biosynthesis, ornithine => arginine
Isoleucine biosynthesis, threonine => 2-oxobutanoate => isoleucine
Leucine biosynthesis, 2-oxoisovalerate => 2-oxoisocaproate
Pyruvate oxidation, pyruvate => acetyl-CoA
F-type ATPase, prokaryotes and chloroplasts
C1-unit interconversion, prokaryotes
Biotin biosynthesis, pimeloyl-ACP/CoA => biotin
Phosphatidylethanolamine (PE) biosynthesis, PA => PS => PE
Fatty acid biosynthesis, elongation
Pyrimidine ribonucleotide biosynthesis, UMP => UDP/UTP,CDP/CTP
Guanine ribonucleotide biosynthesis, IMP => GDP,GTP
Adenine ribonucleotide biosynthesis, IMP => ADP,ATP
Inosine monophosphate biosynthesis, PRPP + glutamine => IMP
Ornithine biosynthesis, glutamate => ornithine
Shikimate pathway, phosphoenolpyruvate + erythrose-4P => chorismate
Cysteine biosynthesis, serine => cysteine
Valine/isoleucine biosynthesis, pyruvate => valine / 2-oxobutanoate => isoleucine
Proline biosynthesis, glutamate => proline
Citrate cycle, first carbon oxidation, oxaloacetate => 2-oxoglutarate
PRPP biosynthesis, ribose 5P => PRPP
Glycolysis (Embden-Meyerhof pathway), glucose => pyruvate
Glycolysis, core module involving three-carbon compounds
C5 isoprenoid biosynthesis, non-mevalonate pathway
NAD biosynthesis, aspartate => quinolinate => NAD
Citrate cycle (TCA cycle, Krebs cycle)
Citrate cycle, second carbon oxidation, 2-oxoglutarate => oxaloacetate
Uridine monophosphate biosynthesis, glutamine (+ PRPP) => UMP
Siroheme biosynthesis, glutamyl-tRNA => siroheme
Tryptophan biosynthesis, chorismate => tryptophan
Pantothenate biosynthesis, valine/L-aspartate => pantothenate
Lysine biosynthesis, DAP dehydrogenase pathway, aspartate => lysine
Lysine biosynthesis, DAP aminotransferase pathway, aspartate => lysine
Nucleotide sugar biosynthesis, glucose => UDP-glucose
Tetrahydrofolate biosynthesis, GTP => THF
NADH:quinone oxidoreductase, prokaryotes
Methionine biosynthesis, aspartate => homoserine => methionine
Cobalamin biosynthesis, cobyrinate a,c-diamide => cobalamin
Gluconeogenesis, oxaloacetate => fructose-6P
KDO2-lipid A biosynthesis, Raetz pathway, non-LpxL-LpxM type
Histidine biosynthesis, PRPP => histidine
KDO2-lipid A biosynthesis, Raetz pathway, LpxL-LpxM type
Thiamine biosynthesis, prokaryotes, AIR (+ DXP/tyrosine) => TMP/TPP
Biotin biosynthesis, BioW pathway, pimelate => pimeloyl-CoA => biotin
Thiamine biosynthesis, archaea, AIR (+ NAD+) => TMP/TPP
Lysine biosynthesis, succinyl-DAP pathway, aspartate => lysine
Biotin biosynthesis, BioU pathway, pimeloyl-ACP/CoA => biotin
dTDP-L-rhamnose biosynthesis
Galactose degradation, Leloir pathway, galactose => alpha-D-glucose-1P
Reductive citrate cycle (Arnon-Buchanan cycle)
Succinate dehydrogenase, prokaryotes
Coenzyme A biosynthesis, pantothenate => CoA
CMP-KDO biosynthesis
Pentose phosphate pathway, non-oxidative phase, fructose 6P => ribose 5P
Pyrimidine deoxyribonucleotide biosynthesis, CDP => dCTP
D-Glucuronate degradation, D-glucuronate => pyruvate + D-glyceraldehyde 3P
Cobalamin biosynthesis, anaerobic, uroporphyrinogen III => sirohydrochlorin => cobyrinate a,c-diamide
Assimilatory sulfate reduction, sulfate => H2S
Methionine degradation
Heme biosynthesis, archaea, siroheme => heme
Reductive pentose phosphate cycle, glyceraldehyde-3P => ribulose-5P
Heme biosynthesis, plants and bacteria, glutamate => heme
Pyrimidine deoxyribonucleotide biosynthesis, UDP => dTTP
Semi-phosphorylative Entner-Doudoroff pathway, gluconate => glycerate-3P
Glycogen degradation, glycogen => glucose-6P
Fatty acid biosynthesis in mitochondria, fungi
Mouse2
MmAkk3
MmAkk2
Akk2650
Akk0880
GP13
GP26
139
Akk2670
MmAkkUCB1
ABMPRH1
MmAkk1
ABMPRH2
YL44
GP19
BSM09
D1015
MucT
Akk2750
Akk0490
GP22
GP24
GP08
Akk2680
GP10
AkkB40
AmV
Estimated Pathway Completion
1
0.8
0.6
0.4
0.2
0

Figure S5: KEGG pathway completeness in our *A. muciniphila* strains.
Predicted KEGG metabolic pathways present in each *A. muciniphila* genome were selected for inclusion here based on having a threshold of 70% completeness of a KEGG Module in at least one strain. The heatmap color scale refers to estimated pathway completion. Numerical values for the heatmap can be found in Table S5.
