# Supplementary material for: Spontaneous episodic inflammation in the intestines of mice lacking HNF4A is driven by microbiota and associated with early life microbiota alterations
Source: mBio. 2023 Aug 1;14(4):e01504-23. doi: 10.1128/mbio.01504-23 (PMC10470520; doi:10.1128/mbio.01504-23)
Supplement: Table S1 — P-values for gnotobiotic experiment. [file mbio.01504-23-s0002.docx]

**Table S1: Comparison of fecal Lcn2 levels for gnotobiotic experimental groups (related to Fig. 4B).** These p-values are for the indicated group comparisons using genotype by colonization status by life stage as the predictor in our repeated measures regression analysis.

| **Condition 1** | **Condition 2** | **p-value** |
| --- | --- | --- |
| CV, *Hnf4a*^ΔIEC^, early life | GF, *Hnf4a*^ΔIEC^, early life | <0.0001 |
| CV, *Hnf4a*^ΔIEC^, late life | GF, *Hnf4a*^ΔIEC^, late life | <0.0001 |
| CV, *Hnf4a*^fl/fl^, early life | GF, *Hnf4a*^fl/fl^, early life | <0.0001 |
| CV, *Hnf4a*^fl/fl^, late life | GF, *Hnf4a*^fl/fl^, late life | 0.054 |
| CV, *Hnf4a^fl/+^;Vil1:Cre+*, early life | GF, *Hnf4a^fl/+^;Vil1:Cre+*, early life | <0.0001 |
| CV, *Hnf4a^fl/+^;Vil1:Cre+*, late life | GF, *Hnf4a^fl/+^;Vil1:Cre+*, late life | 0.022 |
| CV, *Hnf4a*^ΔIEC^, early life | CV, *Hnf4a*^fl/fl^, early life | 0.1045 |
| CV, *Hnf4a*^ΔIEC^, late life | CV, *Hnf4a*^fl/fl^, late life | <0.0001 |
| CV, *Hnf4a*^ΔIEC^, early life | CV, *Hnf4a^fl/+^;Vil1:Cre+*, early life | 0.0007 |
| CV, *Hnf4a*^ΔIEC^, late life | CV, *Hnf4a^fl/+^;Vil1:Cre+*, late life | <0.0001 |
| GF, *Hnf4a*^ΔIEC^, early life | GF, *Hnf4a*^fl/fl^, early life | >0.3 |
| GF, *Hnf4a*^ΔIEC^, late life | GF, *Hnf4a^fl/+^;Vil1:Cre+*, late life | >0.3 |
